# Immune stimulation recruits a subset of pro-regenerative macrophages to the retina that promotes axonal regrowth of injured neurons

**DOI:** 10.1186/s40478-023-01580-3

**Published:** 2023-05-24

**Authors:** Lien Andries, Daliya Kancheva, Luca Masin, Isabelle Scheyltjens, Hannah Van Hove, Karen De Vlaminck, Steven Bergmans, Marie Claes, Lies De Groef, Lieve Moons, Kiavash Movahedi

**Affiliations:** 1 Neural Circuit Development and Regeneration Research Group, Animal Physiology and Neurobiology Division, Department of Biology, Leuven Brain Institute, KU Leuven, Naamsestraat 61, Box 2464, 3000 Louvain, Belgium; 2 Laboratory for Molecular and Cellular Therapy, Vrije Universiteit Brussel, Laarbeeklaan 103, 1090 Brussels, Belgium; 3 Myeloid Cell Immunology Lab, VIB Center for Inflammation Research, Brussels, Belgium; 4 Cellular Communication and Neurodegeneration Research Group, Animal Physiology and Neurobiology Division, Department of Biology, Leuven Brain Institute, KU Leuven, 3000 Louvain, Belgium

## Abstract

The multifaceted nature of neuroinflammation is highlighted by its ability to both aggravate and promote neuronal health. While in mammals retinal ganglion cells (RGCs) are unable to regenerate following injury, acute inflammation can induce axonal regrowth. However, the nature of the cells, cellular states and signalling pathways that drive this inflammation-induced regeneration have remained elusive. Here, we investigated the functional significance of macrophages during RGC de- and regeneration, by characterizing the inflammatory cascade evoked by optic nerve crush (ONC) injury, with or without local inflammatory stimulation in the vitreous. By combining single-cell RNA sequencing and fate mapping approaches, we elucidated the response of retinal microglia and recruited monocyte-derived macrophages (MDMs) to RGC injury. Importantly, inflammatory stimulation recruited large numbers of MDMs to the retina, which exhibited long-term engraftment and promoted axonal regrowth. Ligand-receptor analysis highlighted a subset of recruited macrophages that exhibited expression of pro-regenerative secreted factors, which were able to promote axon regrowth via paracrine signalling. Our work reveals how inflammation may promote CNS regeneration by modulating innate immune responses, providing a rationale for macrophage-centred strategies for driving neuronal repair following injury and disease.

**Supplementary Information:**

The online version contains supplementary material available at 10.1186/s40478-023-01580-3.

## Introduction

The central nervous system (CNS) accommodates various populations of resident macrophages that are critical regulators in brain development, homeostasis and disease [1]. This includes microglia in the brain parenchyma and border-associated macrophages (BAMs) in non-parenchymal border tissues. Microglia continuously survey their microenvironment and interact with neurons to prune synapses, provide neurotrophic factors, remove waste and sense danger [1]. Similarly, BAMs play key roles in supporting healthy brain functions [2]. Thus, resident CNS macrophages are highly specialized cells that play an active role in maintaining healthy brain physiology. Upon inflammation and disease, microglia and BAMs exit their homeostatic state and adopt new transcriptional modules. This has been thoroughly investigated for microglia during neurodegeneration and subsequently in other disease and injury models, where a specific disease-associated microglia (DAM) state has been identified [3–12]. The disease-responses of microglia and BAMs can be beneficial or detrimental depending on the nature and/or stage of the disease. For example, in Alzheimer’s disease DAMs may alleviate amyloid beta (Ab) pathology by compacting amyloid plaques but may also aggravate the disease following the onset of tau pathology [13]. Importantly, inflammation and disease can also result in the recruitment of monocyte-derived macrophages (MDMs) to the CNS. Emerging evidence indicates that recruited MDMs react differently to disease than resident macrophages [14–16]. Recruited MDMs may exert complementary functions and their interplay with resident brain macrophages can shape disease progression [15]. Therefore, inhibiting or promoting monocyte recruitment to the diseased brain may have significant therapeutic implications.

While the key role of brain macrophages in neurodegeneration is firmly established, their contribution to regeneration and repair has remained more elusive. Several studies have reported that brain macrophages can exert neuroprotective activities and contribute to healing and repair following neurodegeneration or CNS injury [17–20]. Different macrophage activation states, ranging from more pro-inflammatory ones that are associated with tissue damage, neuronal loss, axon retraction and demyelination to more anti-inflammatory phenotypes that are linked to neuroprotection [21] and axon regrowth [22, 23], have been suggested to affect repair after CNS injury [24, 25]. Nevertheless, while neuroprotective and regenerative macrophage subtypes are thought to exist, their molecular fingerprint remains poorly characterized.

One powerful model system to investigate the cellular players and the molecules and signalling pathways that contribute to CNS de- and regeneration is the retina-brain connection. Over the past decades, multiple studies, using the retinofugal pathway and the optic nerve crush (ONC) paradigm as a neurodegeneration model, have shown that induction of controlled inflammation in the retina induces retinal ganglion cell (RGC) survival and axonal regrowth in rodents [26–32]. Previous research revealed that an inflammatory stimulation results in infiltration of neutrophils and MDMs in both the vitreous and retina and leads to modulation of resident macrophages, as well as retinal astrocytes and Müller glia [33, 34]. While it is established that an acute inflammatory response is beneficial for survival and axonal regrowth of damaged RGCs, controversy still prevails about which cells, cell states, molecules and pathways are functionally implicated. To investigate the specific contribution of resident and recruited myeloid cells during RGC de- and regeneration, we performed an in-depth characterization of the acute inflammatory response evoked by optic nerve injury, with or without a local inflammatory stimulation using a Toll-like receptor 2 agonist. By combining single-cell RNA sequencing (scRNA-seq) and fate mapping approaches, we elucidated the ontogeny, cell states and functional significance of the resident and recruited macrophage populations that react to RGC degeneration following ONC injury. Our results show that inflammatory stimulation recruits a subset of pro-regenerative MDMs to the retina, which produce secreted proteins that can promote axon regrowth of injured RGCs.

## Results

### Injury to the optic nerve activates resident and recruited myeloid cells in the retina

**Fig. 1:**
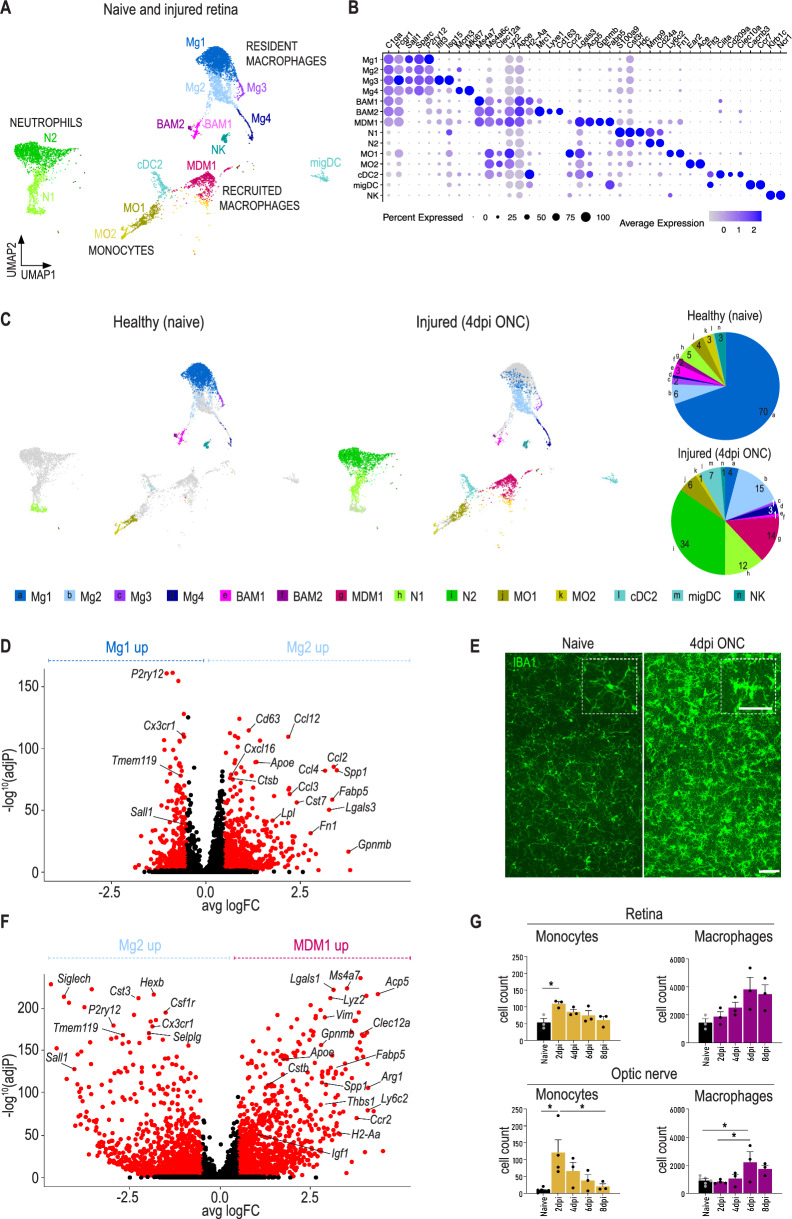
Injury to the optic nerve activates resident and recruited myeloid cells in the retina. **A** UMAP and cluster annotation showing 7128 CD45^+^CD11b^+^ cells isolated from healthy retinas (naive mice) or injured retinas (mice receiving ONC). *BAM* border associated macrophage, *cDC* conventional dendritic cell, *migDC* migratory dendritic cell, *MDM* monocyte-derived macrophage, *Mg* microglia, *MO* monocyte, *N* neutrophils, *NK* natural killer cell. Data originate from retinas pooled from 32 naive mice and 10 ONC mice. **B** Dot plot corresponding to UMAP in (A), showing expression of the indicated genes. Dot size represents the percentage of cells expressing the gene and colour represents its average expression within a cell cluster. **C** UMAP showing 2819 cells of healthy retinas and 4309 cells of injured retinas with a pie chart showing the distribution of different immune cell populations present in the healthy and injured retinas. Numbers in the pie chart represent percentages of the cell subsets. **D** Volcano plot displaying differential expression between Mg2 (injured microglia) and Mg1 (healthy microglia). Genes with adjusted *p*-value < 0.01 and I Log2(FC) I > 1 are shown in red. **E** Retinal wholemounts stained for IBA1 (green) labelling the microglia in the retina of naive mice and mice at 4 dpi ONC. Scale bar 50 µm and 25 µm. Representative images of *n* = 3–4 mice per condition. **F** Volcano plot displaying differential expression between MDM1 and Mg2. Genes with adjusted *p*-value < 0.01 and I Log2(FC) I > 1 are shown in red. **G** Cell counts of monocytes and macrophages at different time points after ONC in the retina and optic nerve, as measured by flow cytometry. Data are shown as mean ± SEM. Repeated measures one-way ANOVA followed by Tukey’s multiple comparisons test, *n* = 3–6 biologically independent samples with retinas from 4 mice pooled per sample. * *P* < 0.05

To investigate the response of myeloid cells in the retina to retinal ganglion cell (RGC) injury, we performed scRNA-seq on CD45^+^CD11b^+^ cells that were sorted from healthy adult retinas or from retinas harvested at 4 days post optic nerve crush (ONC) injury. The majority of CD45^+^CD11b^+^ cells in healthy retinas were microglia, identified based on their high expression of microglial signature genes, including *Sall1*, *Sparc* and *P2ry12* (Fig. 1A, B). Naive retinas also contained small clusters of *Fcgr1*^+^*C1qa*^+^ macrophages that did not express prototypical microglia genes, but exhibited enriched expression of *Ms4a7*, *Ms4a6c* and *Apoe* (clusters BAM1-2) (Fig. 1A, B). Within the brain, this signature is associated with macrophages found in border tissues (border-associated macrophages or BAMs), including the perivascular space [10, 35]. Therefore, these cells may represent retinal BAMs, such as perivascular macrophages. We observed two main clusters that showed differential expression of *Mrc1, Cd163* and *H2-Aa* (Fig. 1B), reflecting BAM heterogeneity in the brain [10]. Other CD11b^+^ cells in the naive retina were neutrophils (*S100a9, Csf3r, Cd24a*), classical monocytes (*Ly6c2, Fn1*), non-classical monocytes (*Ear2*, *Ace*), cDC2 dendritic cells (DCs) (*Flt3*, *Ciita*), migratory DCs (*Flt3, Ccr7*) and natural killer (NK) cells (*Klrb1c, Ncr1*) (Fig. 1A, B).

The immune composition in the retina was clearly altered in mice that underwent an ONC, with the latter showing an increase of peripheral myeloid cells (Fig. 1C). This was most notable for neutrophils, which showed an elevated infiltration in the retina at day 4 post ONC. We also observed a large cluster of cells that expressed both macrophage and neutrophil markers (Additional File 1: Figure S1A, B). These cells may represent macrophage-neutrophil aggregates that formed during in vitro single-cell processing. Alternatively, they may correspond to microglia or macrophages that phagocytosed apoptotic neutrophils in vivo prior to retinal dissection and processing. For reasons of clarity, these cells were excluded from all further analyses.

Clusters Mg1–Mg4 expressed microglial signature genes, including *Sall1*, which is reported to be restricted to embryonically-derived microglia [36–38]. Additionally, Mg1–Mg4 did not express genes related to BAMs or recruited monocyte-derived cells (e.g. *Ms4a7, Clec12a*) [36–38] and were therefore identified as microglia. Interestingly, most retinal microglia from mice that underwent ONC, clustered separately from their counterparts observed in control mice (Fig. 1C), indicating a change in microglial activation status. Microglia from ONC mice were mostly confined to cluster Mg2 that, compared to Mg1, exhibited a downregulation of homeostatic microglial signature genes (*P2ry12, Sall1, Tmem119*) and an induction of prototypical disease-associated microglia (DAM) markers (*Cst7, Lpl, Fabp5*) (Fig. 1D) [3, 39, 40]. This shows that the majority of retinal microglia reacted to RGC axonal injury and that they exhibited gene expression changes that are comparable to those observed for brain microglia responding to neurodegeneration. Mg3 represented a cluster of microglia that expressed interferon (IFN)-induced genes (Additional File 1: Figure S1C) and was observed both in the naive and injured retina. These IFN response microglia are also observed in the healthy brain [41]. We also identified Mg4 as a cluster of proliferating microglia (Additional File 1: Figure S1D), which was most prominent in the post ONC retina (Fig. 1C). The density and activation of microglia in the different layers of the retina (*e.g.* inner and outer plexiform layer) after ONC were further investigated via whole mount retinal staining. Confocal Z-stack images of the inner and outer plexiform layer revealed that microglia shifted from highly ramified cells to bigger more amoeboid cells with retracted processes, indicative for their reactive state (Fig. 1E, Additional File 1: Figure S1E) [42].

Cluster MDM1 represented a subset of *Gpnmb*^hi^ *Fabp5*^hi^ macrophages that clustered distal from microglia and BAMs (Fig. 1A, B, Additional File 1: Figure S1F) and was restricted to the post ONC retina (Fig. 1C). Differential gene expression analysis showed an absence of microglial signature genes in these cells, coupled to a high expression of genes associated with monocyte-derived macrophages (MDMs), including *Ms4a7, Lyz2 *and* Clec12a* (Fig. 1F). Therefore, this cluster may represent MDMs that were recruited to the retina following ONC. In line with this, we observed that microglia from the injured retina exhibited an elevated expression of the monocyte chemoattractant *Ccl2* (Fig. 1D). Furthermore, quantification of monocyte and macrophage infiltration in the retina at days 2, 4, 6 and 8 post ONC via flow cytometry, revealed a transient increase in the number of monocytes, while macrophage numbers peaked at later time points (Fig. 1G). A significant increase in the number of monocytes was also observed in the injured optic nerve (Fig. 1G). This shows that monocytes are attracted to both the retina and optic nerve following ONC injury, which is in line with MDM1 representing newly-recruited monocyte-derived cells. Interestingly, the MDM1 cluster exhibited a high expression of *Apoe, Spp1* and *Igf1* (Additional File 1: Figure S1F), which have been reported to be key genes involved in promoting RGC regeneration following axonal injury [43]. This suggests that recruited MDMs can attain activation states that may promote RGC survival and/or axon regeneration. However, as no axonal outgrowth is observed following ONC, this response is potentially insufficient, or the number of recruited cells too low to induce regeneration.

### Inflammatory stimulation upon ONC recruits monocyte-derived cells that exhibit long-term engraftment in the retina and promote axonal regeneration

As we observed the expression of potential pro-regenerative genes in MDMs, we hypothesized that the reported regeneration of RGC axons following inflammatory stimulation may in part be driven by an increased recruitment of monocyte-derived cells in the retina. To investigate this, we combined ONC injury with intravitreal injection of the Toll-like receptor 2 agonist Pam3Cys combined with cAMP (P3C) and confirmed that this type of inflammatory stimulation induced axonal regrowth of the damaged RGCs (Additional File 2: Figure S2A, B) [28].

**Fig. 2:**
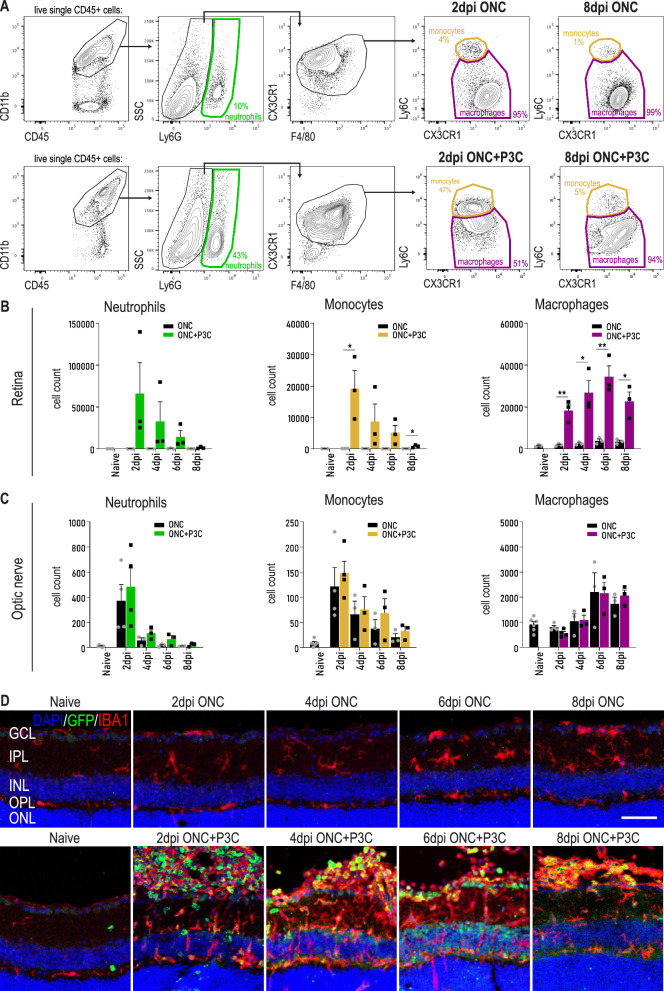
Inflammatory stimulation upon optic nerve injury mobilizes the infiltration of monocyte-derived cells. **A** Representative flow cytometry plots showing the gating strategy used to identify neutrophils (Cx3cr1^low^, Ly6G^high^), monocytes (Ly6C^high^, Ly6G^−^) and macrophages (Cx3cr1^high^, Ly6C^low^) in the retina and optic nerve of mice subjected to ONC and ONC combined with P3C treatment. Example plots were taken from retinas of mice at 2 dpi ONC and at 2 dpi ONC + P3C. Cells were pre-gated as live, single CD45^+^ cells. **B** Cell counts of neutrophils, monocytes and macrophages at different time points after ONC (black) and ONC + P3C (coloured) in the retina, as measured by flow cytometry. Counts of the monocytes and macrophages after ONC correspond to the data shown in Fig. 1G. Data are shown as mean ± SEM. Statistical significance between ONC and ONC + P3C was evaluated via an unpaired *t*-test. *n* = 3–6 biologically independent samples with retinas from 4 mice pooled per sample. * *P* < 0.05 ** *P* < 0.01 **C** Cell counts of neutrophils, monocytes and macrophages at different time points after ONC (black) and ONC + P3C (coloured) in the optic nerve, as measured by flow cytometry. Counts of the monocytes and macrophages after ONC correspond to the data shown in Fig. 1G. Statistical significance between ONC and ONC + P3C was evaluated via an unpaired *t*-test. *n* = 3–6 biologically independent samples with optic nerves from 4 mice pooled per sample. **D** Retinal cryosections of *Lyz2-GFP* (green) mice stained for IBA1 (red). Sections are counterstained with DAPI (blue). Scale bar 50 µm. *GCL* ganglion cell layer, *IPL* inner plexiform layer, *INL* inner nuclear layer, *OPL* outer plexiform layer, *ONL* outer nuclear layer. Representative images of *n* = 3 mice per condition

To assess the kinetics of peripheral myeloid cell infiltration, we performed flow cytometric analysis of the retina and optic nerve from C57BL/6 mice at days 2, 4, 6 and 8 post ONC or ONC + P3C. P3C treatment induced a large increase in the number of recruited neutrophils and monocytes in the retina, representing a ~ 360-fold and ~ 180-fold increase at day 2, respectively (Fig. 2A, B). Neutrophil infiltration was very transient, with most cells disappearing by day 8 post treatment (Fig. 2B). Monocyte recruitment was similarly transient, but these cells gradually differentiated into macrophages, as reflected by a loss of Ly6C and an increase in CX3CR1 expression (Fig. 2A). Coupled to this we observed a strong increase in the number of retinal macrophages, which peaked around day 6 post treatment (Fig. 2B). In contrast, the number of monocytes and macrophages in the optic nerve was not significantly different between the ONC and ONC + P3C conditions (Fig. 2C). This indicates that monocyte influx following intravitreal P3C injection is restricted to the retina. To spatially localize the infiltrating monocyte-derived cells within the retina and vitreous, an immunostaining for IBA1 was performed on retinal sections of *Lyz2*-GFP mice subjected to ONC or ONC + P3C (Fig. 2D). As shown in our scRNA-seq data, *Lyz2* is highly expressed in monocytes and monocyte-derived cells (Additional File 1: Figure S1F). IBA1^+^GFP^+^ cells thus likely represent monocytes and MDMs, although we cannot rule out that a fraction of microglia may also upregulate *Lyz2* following ONC and P3C treatment. P3C treatment induced a strong infiltration of IBA1^+^GFP^+^ cells that at day 2 were mostly observed in the vitreous and showed a round shape, indicative of monocytes (Fig. 2D). Over time, these cells gradually changed their morphology, adopting a more macrophage-like shape, and infiltrated the ganglion cell layer, inner plexiform layer, inner nuclear layer and the outer plexiform layer of the retina (Fig. 2D).

**Fig. 3:**
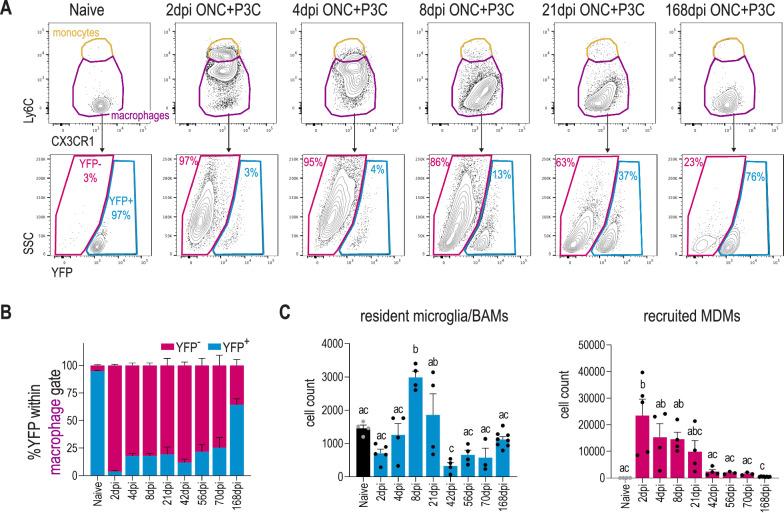
The infiltrating monocyte-derived cells exhibit a long-term engraftment in the retina. **A** Representative flow cytometry plots showing the gating strategy for distinguishing resident macrophages (YFP^+^) from recruited monocyte-derived counterparts (YFP^−^) based on YFP expression in retinas of *Cx3cr1*^*CreER*^:*R26-YFP* mice at various timepoints ranging from 2 to 168 dpi ONC + P3C. Cells were pre-gated as live single CD11b^+^CD45^+^ Ly6G-CX3CR1^+^ F480^+^ as shown in Fig. 2A. **B** Compiled flow cytometry data showing the percentage of YFP^+^ and YFP^−^ cells in the macrophage gate (live single CD11b^+^CD45^+^ Ly6G-CX3CR1^+^ F480^+^) at different time points after ONC + P3C ranging from 2 to 168 dpi ONC in the retina. Data are shown as mean ± SEM. *n* = 3–8 biologically independent samples containing 1 retina from 1 mouse. **C** Cell count of YFP^+^ resident macrophages (microglia and BAMs) and YFP^−^ recruited MDMs at different time points after ONC + P3C ranging from 2 to 168 dpi ONC in the retina, as measured by flow cytometry. Data are shown as mean ± SEM. Repeated measures one-way ANOVA followed by Tukey’s multiple comparisons test, statistical significance between different time points is indicated using different letters: conditions that share the same letter are not significantly different, while conditions with different letters are significantly different from each other. *n* = 3–8 biologically independent samples with 1 retina from 1 mouse

The increased number of macrophages observed in P3C treated mice may be driven by an increased recruitment of monocytes and/or by an expansion of resident microglia/BAMs. To distinguish between these possibilities, we performed fate mapping using the *Cx3cr1*^CreER^: R26-YFP model. Upon tamoxifen treatment in *Cx3cr1*^CreER^: R26-YFP mice, 95 ± 1% of retinal macrophages were YFP labelled (Fig. 3A, B). Four weeks following tamoxifen administration, when YFP labelling in monocytes is lost [44], mice received ONC + P3C treatment. Retinas were processed for flow cytometry at various time points, ranging from 2 to 168 days post treatment. We were able to distinguish resident microglia/BAMs from recruited MDMs based on their differential YFP expression (Fig. 3A). This confirmed that the strong increase in retinal macrophages upon P3C treatment was driven by the recruitment and differentiation of monocytes (Fig. 3B, C). While the number of recruited macrophages progressively decreased, a substantial fraction showed long-term engraftment. At 70 days post treatment 75 ± 9% of macrophages were still recruited YFP^−^ cells (Fig. 3B). However, at 168 days post treatment, the percentage of YFP^−^ retinal macrophages had dropped to 30 ± 2%. Together, these data suggest that while a fraction of recruited MDMs were long-lived, their engraftment was still transient as they were progressively lost and/or replaced by embryonic microglia.

**Fig. 4:**
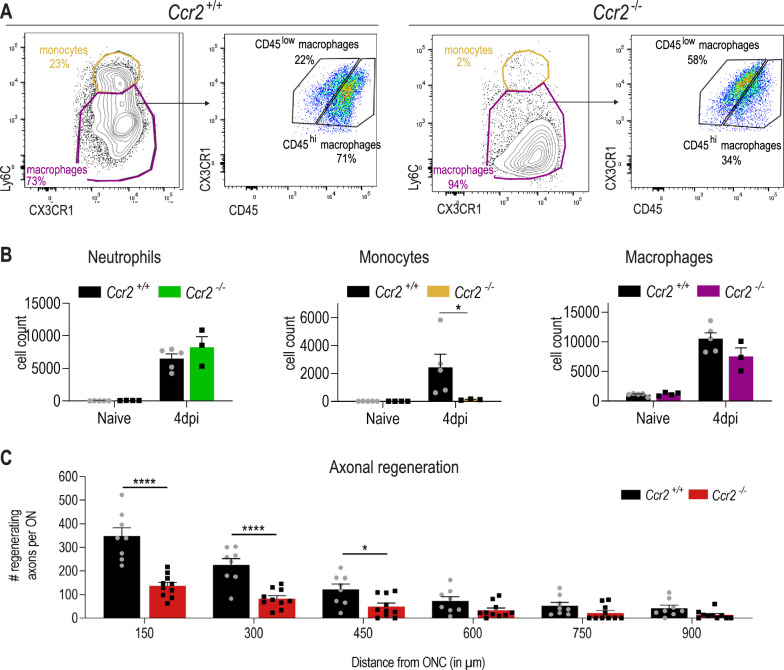
Recruited monocyte-derived macrophages that infiltrate the retina upon inflammatory stimulation promote axonal regeneration. **A** Representative flow cytometry plots of cells from the retina of *Ccr2*^+*/*+^ and *Ccr2*^*−/−*^ mice at 4 dpi ONC + P3C. Cells were pre-gated as shown in Fig. 2A. Percentages of monocytes and macrophages and of CD45^hi^ and CD45^low^ macrophages are shown at 4 dpi ONC + P3C. **B** Cell counts of neutrophils, monocytes and macrophages at 4 dpi ONC + P3C in the retina of *Ccr2*^+*/*+^ (black) and *Ccr2*^*−/−*^ (coloured) mice, as measured by flow cytometry. Data are shown as mean ± SEM. Statistical significance between CCR2^+/+^ and CCR2^−/−^ was evaluated via the Mann–Whitney test. *n* = 3–5 biologically independent samples with 1 retina from 1 mouse. * *P* < 0.05. **C** Quantification of axonal regeneration on longitudinal cryosections of the optic nerve of *Ccr2*^+*/*+^ and *Ccr2*^*−/−*^ mice at 14 dpi after ONC + P3C, analysed at various distances, starting from 150 µm after the ONC lesion site. Data are shown as mean ± SEM. Repeated measures two-way ANOVA followed by Tukey’s multiple comparisons test. *n* = 8–10 mice per condition. ****p < 0.0001 and *p < 0.5

Next, we aimed to investigate whether MDMs that infiltrate the retina upon inflammatory stimulation promote axonal regeneration. Therefore, we performed ONC + P3C treatment in *Ccr2-*deficient mice, which are known to have low numbers of blood monocytes [45]. Flow cytometry at day 4 post treatment confirmed that the number of infiltrating monocytes was strongly reduced in ONC + P3C treated *Ccr2-*deficient mice as compared to *Ccr2*^+*/*+^ controls, while infiltration of neutrophils was unaltered (Fig. 4A, B). Furthermore, while most macrophages in ONC + P3C treated *Ccr2*^+*/*+^ retinas were CD45^hi^, in *Ccr2*^*−/−*^ retinas they were CD45^low^, indicative of microglia (Fig. 4A). These data thus reveal that in *Ccr2*^*−/−*^ mice the recruitment of monocyte-derived macrophages to the retina was strongly impaired. Importantly, *Ccr2*^−/−^ mice also showed a significantly reduced number of CTB^+^ regenerating axons (Fig. 4C). This indicates that monocyte-derived macrophages that are recruited to the retina upon P3C treatment, promote axonal regeneration of injured RGCs.

### Nerve injury combined with inflammatory stimulation results in the hyperactivation of microglia which remain distinct from recruited monocyte-derived macrophages

**Fig. 5:**
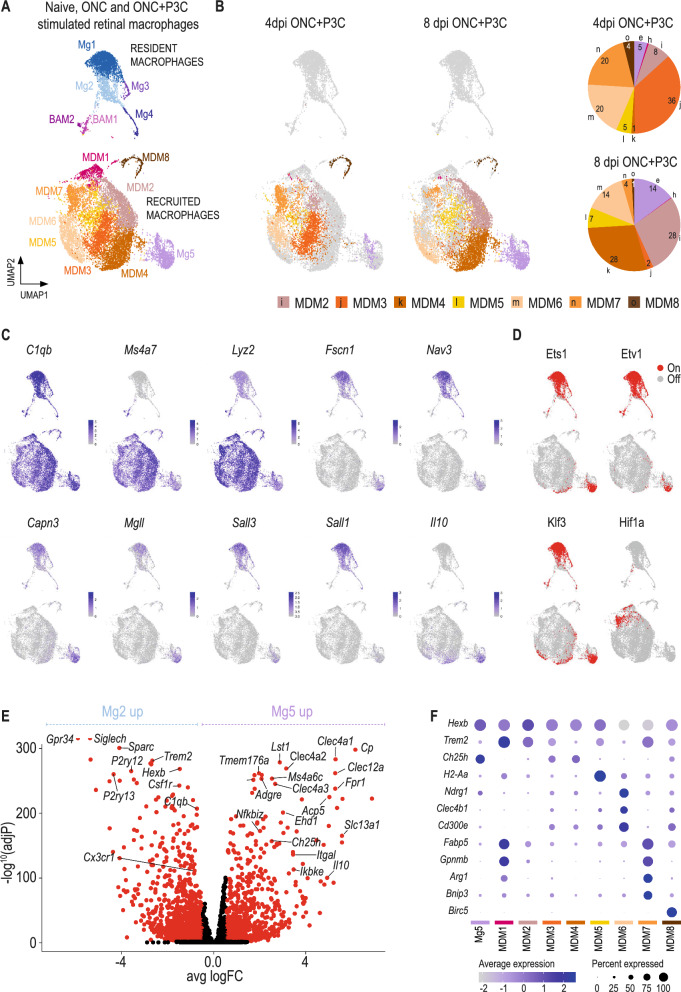
Nerve injury combined with inflammatory stimulation results in the hyperactivation of microglia which remain distinct from recruited monocyte-derived macrophages. **A** UMAP and cluster annotation showing 14,963 macrophages of healthy, injured (4 dpi ONC) and regenerating (4 and 8 dpi ONC + P3C) retinas. BAM, border associated macrophage, MDM: monocyte-derived macrophage, Mg: microglia. Data originate from retinas pooled from 32 naive mice, 10 ONC mice, 4 ONC + P3C 4 dpi mice and 4 ONC + P3C 8dpi mice. **B** UMAP showing 3974 macrophages of regenerating retinas at 4 dpi ONC + P3C and 6957 macrophages of regenerating retinas at 8 dpi ONC + P3C with a pie chart showing the distribution of different macrophage populations present in the injured + P3C treated retinas. Numbers in the pie chart are indicating percentages of macrophage subsets. **C** Corresponding UMAPs revealing the expression of signature genes that differentiate between the multiple macrophage populations. The colour (grey, low expression; purple, high expression) represents the expression profile in the macrophage clusters. **D** Corresponding UMAPs showing the bimodal regulon activity of specific microglia and monocyte-derived regulons, with red dots indicating an active regulon in the corresponding cells. Regulon here refers to a module of co-expressed genes together with their corresponding transcription factor. **E** Volcano plot displaying differential expression between Mg5 and Mg2, Genes with adjusted *p*-value < 0.01 and I Log2(FC) I > 1 are shown in red. **F** Corresponding dot plot to the UMAP plot in Figure **A**, showing the expression of subset-specific genes, with the dot size representing the percentage of cells expressing the gene and the colour representing its average expression within a cluster

To profile the cell states and heterogeneity of macrophages in the retina following inflammatory stimulation, we performed scRNA-seq on CD45^+^CD11b^+^ cells sorted from the retina at day 4 and 8 post ONC + P3C treatment. The obtained data were combined with those of the naive and day 4 post ONC retina in a single dataset (Fig. 5A). Additionally, to obtain insights into the transcription factors and gene regulatory networks that shape the activation state of macrophages, we performed single-cell regulatory network inference and clustering (SCENIC) analysis on all microglia and macrophage clusters [46, 47].

P3C treatment resulted in a large population of *C1qb*^+^ macrophages within the retina (Fig. 5A–C). Most clusters expressed high levels of *Ms4a7* and *Lyz2* (Fig. 5C), suggesting that these cells were recruited monocyte-derived cells (MDM2-MDM8). *Ms4a7* and *Lyz2* were low in cluster Mg5, which also showed signature expression of *Fscn1*, *Nav3*, *Capn3* and *Mgll*, genes that were shared with naive and ONC-only microglia (Fig. 5C). Mg5 was also the only macrophage cluster within the ONC + P3C-treated retinas that exhibited *Sall3* and *Sall1* expression (Fig. 5C), genes known to be highly restricted to embryonic microglia. SCENIC analysis showed that the microglia-associated regulons Ets1, Etv1 and Klf3 were also active in Mg5 (Fig. 5D). Together, this suggests that Mg5 represented microglia, while MDM2-8 were recruited macrophages that remained transcriptionally distinct from microglia. Cluster Mg5 represented 3% and 13% of the profiled CD11b^+^ cells at day 4 and 8 post ONC + P3C, respectively (Additional File 3: Figure S3). This was in line with our previous fate mapping data, where we observed 4 ± 1% and 16 ± 2% YFP^+^ microglia within CD11b^+^ cells at 4 and 8 days post treatment, respectively, as observed via flow cytometry in ONC + P3C treated *Cx3cr1*^CreER^:*R26-YFP* mice. However, as we also observed enriched expression of genes that are related to MDMs or BAMs, such as *Clec12a*, *Clec4a1* and *Itgal* [10] (Fig. 5E), we cannot rule out that part of the Mg5 cluster is monocyte or BAM-derived. The putative Mg5 microglia from ONC + P3C treated retinas showed many differentially expressed genes when compared to the Mg2 DAM cluster from ONC-only retinas (Fig. 5E), suggesting a hyperactivation upon P3C treatment. This included a further downregulation of homeostatic signature genes in Mg5 as compared to Mg2 and an induction of genes related to inflammatory activation, as highlighted by gene ontology (GO) analysis (Additional File 4: Figure S4). Mg5 cells also showed robust expression of the anti-inflammatory cytokine *Il10* (Fig. 5C). This suggests that in the retina, microglia can produce IL10 following nerve injury combined with TLR2 stimulation, which is not observed in brain microglia upon peripheral LPS challenge [48].

MDMs from ONC + P3C treated retinas (MDM2-7) were distinct from MDMs observed in the ONC only condition (MDM1), indicating that P3C administration not only affected the level of MDM recruitment, but also altered their molecular state. Furthermore, gene expression in ONC + P3C MDMs was dynamic in time, showing transcriptional divergence between cells profiled at day 4 versus day 8 post treatment (Fig. 5B). Interestingly, MDMs from ONC + P3C retinas exhibited a high level of heterogeneity (clusters MDM2-8). A fraction of these MDMs showed enriched *Hexb* expression (MDM2-5), and these cells could be further subdivided into a *Trem2*^hi^ (MDM2), *Ch25h*^hi^ (MDM3-4) and *H2-Aa*^hi^ cluster (MDM5) (Fig. 5F). Within the *Hexb*^low^ macrophages, MDM6 expressed *Ndrg1, Clec4b1 and Cd300e*, while cells in MDM7 were *Trem2*^+^ and showed an enriched expression of genes involved in phagocytosis and lipid metabolism, including *Fabp5* and *Gpnmb* (Fig. 5F). The latter genes were also enriched in MDMs from the ONC-only retinas (MDM1). Additionally, MDM7 expressed genes that are associated with hypoxia or HIF1a signalling, including *Arg1* and *Bnip3* [49]. An active HIF1 regulon in MDM7 was also identified via SCENIC (Fig. 5D). MDMs also exhibited proliferative potential as represented by the MDM8 cluster.

### Identification of a pro-regenerative gene signature in recruited monocyte-derived macrophages

**Fig. 6:**
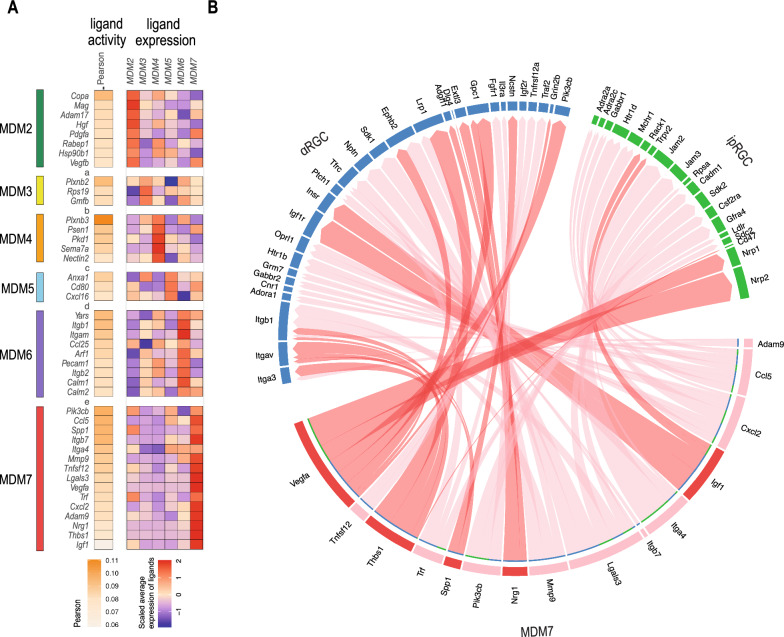
Identification of a pro-regenerative gene signature in monocyte-derived macrophages. **A** Top ligands of each macrophage cluster were selected and ranked based on their ligand activity values. Each ligand was assigned to a cluster if it was expressed highest in this cluster compared to the remaining sender clusters. The colour (white, low expression; orange, high expression) represents the predicted activity of the ligands. The expression of the top ligands in each cluster is shown with the colour (blue, low expression; red, high expression) representing the scaled average expression in the corresponding cluster. **B** Circle plot of potential ligand-receptor pairs, that shows the links between predicted ligands from MDM cluster 7 with their associated receptors found on alpha- and intrinsically photosensitive retinal ganglion cells

We wished to assess the nature of the crosstalk that exists between macrophages and injured RGCs in ONC + P3C retinas and to identify important molecules and pathways for axonal regrowth. Hereto, we relied on the dataset from Tran et al., who profiled RGCs from naive and injured retinas at various time points post ONC via scRNA-seq [50]. We merged their dataset with ours and relied on the NicheNet algorithm [51] to screen for potential ligand-receptor interactions between macrophages ("senders") and RGCs ("receivers"). We focused our analysis on intrinsically photosensitive RGCs (ipRGCs) and αRGCs [50], which are the subclasses most resilient to ONC injury and known to have regenerative capacity [50, 52]. NicheNet was used to predict the ligand-receptor interactions that may drive the gene expression changes observed in healthy versus injured RGCs at day 4 post ONC. Top ligands from the various macrophage clusters were selected and ranked based on their Pearson values between a ligand’s target predictions and the observed transcriptional response within the ONC + P3C retinas (Fig. 6A), together with an overview of the potential receptors (Additional File 5: Figure S5A) and affected target genes in RGCs (Additional File 5: Figure S5B). Most ligand-receptor interactions were predicted for the MDM6 and MDM7 sender clusters (Additional File 5: Figure S5C), highlighting the crosstalk between these macrophage subsets with the injured RGCs. Interestingly, NicheNet identified the ligands *Spp1, Thbs1, Vegfa* and *Igf1* in MDM7 (Fig. 6B). These are secreted proteins that are known to promote RGC survival and/or axon regeneration following ONC injury [52–57]. Another predicted ligand was *Nrg1*, which is involved in axon regeneration following peripheral nerve injury [58]. Besides these predicted ligands, MDM7 also showed enriched gene expression for other secreted factors known to be involved in either RGC or peripheral tissue regeneration, including *Slpi*, *Sdc1* and *Fstl1* (Additional File 6: Figure S6A) [59–64]*.* These ligands are currently not included in the NicheNet ligand-target database and thus cannot be predicted, but their expression further suggests a pro-regenerative signature in MDM7. This pro-regenerative phenotype may underlie the macrophage-mediated axonal regeneration that we observe in P3C treated retinas. Previous work has suggested the involvement of oncomodulin and SDF1/CXCL12 in monocyte/macrophage mediated RGC regeneration [65–69]. However, gene expression of *Onc* and *Cxcl12* was not or hardly detected in the CD11b^+^ cells from our dataset (Additional File 6: Figure S6B).

### The pro-regenerative factors identified in monocyte-derived macrophages can promote axon regeneration via paracrine signalling

**Fig. 7:**
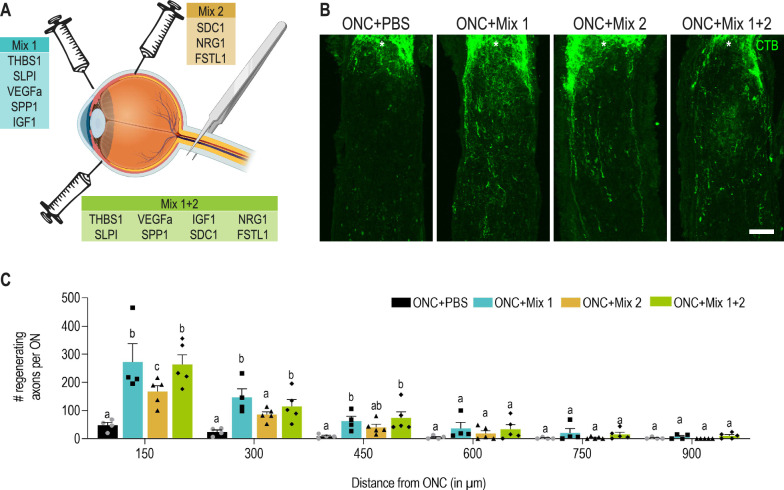
The pro-regenerative factors identified in monocyte-derived macrophages can promote axon regeneration via paracrine signalling. **A** Schematic overview of the experimental setup of the different recombinant protein mixes intravitreally injected in the eye. **B** Representative images of regenerating axons that were CTB-traced on longitudinal cryosections of the optic nerve of mice at 14 dpi ONC and intravitreally injected with PBS, mix 1, mix 2 and mix 1 + 2. The ONC site is indicated with an asterisk. Scale bar 100 µm. **C** Quantification of axonal regeneration on longitudinal cryosections of the optic nerve of mice at 14 dpi ONC and intravitreal injection of PBS, mix 1, mix 2 and mix 1 + 2. Axonal number was counted at various distances starting at 150 µm from the ONC lesion site. Data are shown as mean ± SEM. Repeated measures two-way ANOVA followed by Tukey’s multiple comparisons test, statistical significance between different conditions at the same distance is indicated with different letters: conditions that share the same letter are not significantly different, while conditions with different letters are significantly different from each other, *n* = 4–5 mice per condition

Pro-regenerative factors such as THBS1, SPP1 or IGF1 have been described in the context of autocrine signalling, as these proteins are produced by surviving RGCs [52, 53, 56]. We hypothesized that the secretion of these factors by macrophages may also contribute to RGC regrowth via paracrine signalling. To investigate this, we composed two mixtures of recombinant proteins based on the pro-regenerative signature identified in cluster MDM7. Mix 1 (THBS1, SLPI, VEGFA, SPP1 and IGF1) consisted of secreted proteins reported to induce axonal regeneration in the central nervous system. Mix 2 contained factors that have been shown to stimulate regeneration upon their release in the periphery (SDC1, NRG1 and FSTL1), but have not yet been assessed in the context of RGC axonal regrowth. The individual mixes or the combination of both were injected in the vitreous immediately after ONC, and at day 3 and 7 post ONC (Fig. 7A). Control mice received intravitreal injections of PBS. Axonal regeneration was assessed by quantifying the number of CTB^+^ regrowing axons at day 14 post ONC on longitudinal optic nerve sections (Fig. 7B, C). Interestingly, both mixes of recombinant proteins induced axonal regeneration in the optic nerve. The largest increase in the number of regenerating axons was observed for Mix 1, while a lower but still significant axonal regrowth was observed for Mix 2. These results show that the recombinant proteins within mix 1 and 2 can stimulate the regeneration of RGCs when injected in the vitreous. This implies that the secretion of these pro-regenerative factors by MDMs can contribute to axonal regeneration via paracrine signalling in RGCs.

## Discussion

The nature of the cell populations, cellular states, as well as the molecules and signalling pathways that underly inflammation-induced axonal regrowth have remained elusive [33]. Our work now further highlights how inflammation and CNS regeneration are intertwined and provides evidence for a key role played by recruited MDMs.

Similar to the brain, the retina contains yolk-sac-derived microglia that self-renew and rely on CSF1 or IL34 for their maintenance [40]. Our single-cell transcriptomic profiling confirmed that homeostatic microglia are the predominant myeloid cells in the healthy retina and also revealed subsets of retinal BAMs that likely correspond to perivascular macrophages [35]. Upon ONC-induced RGC degeneration, resident macrophages changed their expression profile by downregulating homeostatic genes and upregulating genes related to inflammatory activation. Notably, the specific neurodegenerative expression profile of retinal microglia in our nerve crush injury model was similar to the expression profile of retinal DAMs observed under conditions of light-damage-induced photoreceptor degeneration [40] or in glaucoma models [70]. It also resembles the molecular signature of brain DAMs observed in amyloid models of Alzheimer’s disease [3, 10, 71] and other pathological conditions of the CNS [5, 6, 8, 15, 72–74]. Therefore, the DAM phenotype is broadly similar in the brain and retina and across multiple etiologically distinct diseases or injury models. Furthermore, it is also not strictly disease-associated, as a similar cell state is also observed for microglia from healthy young mice that engulf myelin [9] or apoptotic neurons [4, 7] and for non-parenchymal microglia that live on the choroid plexus epithelium [10]. Therefore, microglia seem to react in a similar way to many homeostatic disturbances. This may be a common feature of tissue-resident macrophages, as their highly specialized phenotypes require tissue-imprinting that may limit their plasticity towards inflammatory insults [16]. The functional significance of the DAM response is dependent on the nature of the disturbance. In the retina it can be protective during photoreceptor degeneration [40] but detrimental for RGC survival during glaucoma [70]. However, local P3C treatment did significantly alter microglial activation beyond the DAM state, further reducing homeostatic signature genes and driving inflammatory activation. This suggests that strong TLR signalling induced by local P3C injection led to a hyperactivation of microglia. However, as these cells also expressed genes related to recruited MDMs, we cannot rule out that they were partly monocyte derived. It will be interesting to further assess the ontogeny and functional significance of these cells during RGC regeneration in follow-up studies.

Monocytes that are recruited during disease may react differently to the local inflammatory cues as compared to resident macrophages. Previous myeloid cell fate mapping studies, performed after ONC injury [35] and in other retinal injury models, have highlighted a role for recruited MDMs [14, 75–77]. We observed that inflammatory treatment resulted in a strong recruitment of MDMs into the retina. ScRNA-seq analysis revealed that MDMs were transcriptionally distinct from resident microglia and BAMs and exhibited transcriptional heterogeneity. Our data thus confirm that recruited MDMs exhibit substantial plasticity and show disease-specific adaptation. As inhibiting MDM recruitment also impaired axonal outgrowth, this revealed the importance of recruited MDMs in promoting RGC regeneration. An important contribution of myeloid cells to inflammation-induced optic nerve regeneration has long been debated, with conflicting views [25, 26, 65, 78, 79]. A possible explanation is the differential effect of resident versus recruited macrophages. Further dissecting the role of individual macrophage subsets or cell states will provide additional insights into the multifaceted role of innate immunity in neurodegeneration versus protection and repair. In animal models of acute spinal cord injury [20] and brain ischemia [19], MDMs were observed to exert a neuroprotective role and to facilitate repair, by displaying multiple functions including anti-inflammatory [18, 20, 80] and scar degrading roles [21, 81], as well as the ability to support axonal growth [20, 65, 80, 82, 83]. Although our data highlight the importance of recruited MDMs, we do not exclude that also other immune or non-immune cell types contribute to the observed inflammation-induced axonal regeneration. For instance, a recent study identified a subset of immature neutrophils with neuroprotective and regenerative properties [84]. Furthermore, reactive macroglia (*i.e.* astrocytes and Müller glia) may also add to the inflammation-enhanced axonal regeneration. Evidence indeed exists for reciprocal interactions between innate immune cells and macroglia in shaping the CNS response to injury and disease [35, 85–88].

One of the first myeloid-cell-derived molecules reported to play a central role in RGC axonal regrowth is oncomodulin. This small calcium-binding protein is reported to be secreted by macrophages and/or neutrophils during Zymosan-driven ocular inflammation and to induce RGC survival and axonal regeneration via a Ca^2+^/calmodulin kinase-dependant pathway [65–68]. Furthermore, SDF1, also known as CXCL12, expressed by infiltrating monocytes/MDMs was reported to enhance oncomodulin activity [69]. Upon ONC + P3C treatment we did not identify *Ocm* or *Cxcl12* gene expression in resident or recruited macrophages. We did identify a cluster of MDMs showing enriched expression of multiple genes encoding proteins that have been shown to exert pro-regenerative effects in the CNS and are known to be secreted. One of the most highly expressed genes encodes for thrombospondin 1 (THBS1), a protein that is well-known to mediate axon regeneration of RGCs in an autocrine fashion [53]. Bray et al. showed that the observed effect of THBS1 depends on syndecan 1 (SDC1), a THBS1-binding protein (Bray et al. 2019). Autocrine *Sdc1* signalling has also been reported to mediate axon regrowth in the mouse PNS [59]. Other secreted proteins expressed after the ONC + P3C treatment and known to promote regeneration of RGCs include secretory leukocyte protease inhibitor SLPI, osteopontin (SPP1) and insulin growth factor 1 (IGF1) [52, 56, 63]. Moreover, the putatively regenerative MDMs also expressed genes for secreted proteins that are known to be pro-regenerative in the PNS, such as VEGF [54], NRG1 [89] and FSTL1 [64]. Our study now reveals that these previously identified pro-regenerative molecules are also produced by a specific subcluster of MDMs in the regenerating retina and that these factors can induce axonal regrowth of injured RGCs via non-cell autonomous paracrine signalling. SCENIC analysis identified HIF1A as a putative transcription factor that was driving this cell state. It will be important to further identify the microenvironmental signals and the gene regulatory networks that control the pro-regenerative phenotype of MDMs in future studies. This may pave the road for macrophage-centred strategies for inducing and promoting neuroprotection and repair following injury and disease.

## Material and methods

### Animals

All experiments were performed using a combination of male and female 8–12-week-old mice of following strains: C57BL/6 wild-type, *Lyz2-*GFP [90]*, Cx3cr1*^CreER^ [91]*,* *R26-YFP *[92] and* Ccr2*^*−/−*^ [45] mice, as outlined in Additional File 7: Table S1. All animal experiments were approved by the Institutional Ethical Committees for Animal Experimentation of KU Leuven and the Vrije Universiteit Brussel and were conducted in strict accordance with the European and Belgian legislation.

### Tamoxifen treatment

Three-to-four-week-old anesthetized *Cx3cr1*^*CreER*^*: R26-YFP* mice were treated with tamoxifen (Sigma Aldrich, 20 mg/ml dissolved in corn oil (Sigma Aldrich)), which was injected subcutaneously near the fore- and hind limbs (4 × 50 µl). These injections were repeated three times at 48 h intervals.

### Intraorbital optic nerve crush model

Optic nerve crush (ONC) was performed as previously described [93, 94]. Briefly, mice were anesthetized by intraperitoneal injection of a mixture of ketamine (Anesketin, Eurovet, 75 mg/kg body weight) and medetomidine (Domitor, Pfizer, 1 mg/kg body weight) diluted in saline (NaCl, Fischer Scientific, 0.9% in H_2_O). and a topical aesthetic ointment (oxybuprocaïnehydrochloride, Unicaïne, Thea Pharma, 0,4%) was applied on the injured eye. An incision in the temporal side of the conjunctiva was made in the left eye. Then, the posterior side of the eye was exposed, allowing visualization of the optic nerve. The exposed optic nerve was crushed approximately 1 mm from the optic nerve head with a cross-action forceps for 5 s. Thereafter, a fundoscopy was performed and animals with signs of ischemia were excluded. Eyes from uninjured mice were used as controls.

### Intravitreal injections

Intravitreal injections were performed as previously described [93, 95]. Briefly, a Hamilton syringe equipped with a 34G Hamilton needle was inserted into the nasal part of the eye of anesthetised mice, at the limbus, under a 45° angle to avoid damage to the lens. To induce an acute inflammatory stimulation, 2 µl of a combination of Pam3Cys (P3C, Sigma Aldrich, 2.5 µg/µl in sterile PBS [96]) and chlorophenylthio-cyclic adenosine monophosphate (CPT-cAMP, cAMP analogue, Sigma-Aldrich, 50 µM in PBS) was injected immediately after the ONC surgery. To trace regenerating RGC axons in the optic nerve, 2 µl of cholera toxin subunit B conjugated to an Alexa Fluor 488 fluorophore (CTB-Alexa488; Sigma Aldrich, 5 µg/µl in sterile PBS containing dimethylsulfoxide (DMSO, Sigma Aldrich, 0,5%)) was injected one day before sacrificing the mice. Recombinant proteins (THBS1, SLPI, VEGFa, SPP1, IGF1, SDC1, NRG1 FSTL1, R&D systems, 1 µg/µl in sterile PBS) were injected 3 × 2 µl at 0, 3 and 7dpi ONC.

### Flow cytometry of myeloid inflammatory cells

Mice were euthanized with an intraperitoneal injection of an overdose of pentobarbital (Dolethal, Vetoquinol, 200 mg/kg body weight) and transcardially perfused with saline to remove all blood. Eyes were harvested and retinas and optic nerves dissected and transferred to Roswell Park Memorial Institute (RPMI) 1640 medium (Gibco). For the retinal samples, the retinal pigment epithelium was detached from the retina, but the vitreous was not removed in order to include the infiltrating immune cells that were localized at the retina-vitreous interface. A single-cell suspension was obtained by mechanical and enzymatic (collagenase I (Worthington, 10 U/ml), collagenase IV (Worthington, 400 U/ml) and DNase I (Worthington, 30 U/ml) diluted in Hank's Balanced Salt Solution (HBSS) medium (Gibco)) dissociation as previously described (3 × 10 min at 37 °C) [10]. Afterwards, these cells were filtered, washed in MACS buffer (HBSS medium (Gibco) supplemented with sterile filtered ethylenediaminetetraacetic acid (EDTA; Duchefa; 2 mM) and heat-inactivated fetal calf serum (FCS, Gibco, 2%)) and blocked with anti-mouse CD16/CD32 (clone 2.4G2, BD Biosciences, 2 µg/µl in MACS buffer). Cells were stained with fluorescent antibodies in MACS buffer. The following antibodies were used: F4/80 (BV421, clone BM8, Biolegend), CD11c (PE/Cy7, BV510, clone N418, Biolegend), Ly6G (FITC, clone 1A8, Biolegend), Cx3cr1 (PE, clone SA011F11, Biolegend), CD11b (PE/Cy7, BV510, clone M1/70, Biolegend), Ly6C (APC, BV421, clone HK1.4, Biolegend), CD45 (APC/Cy7, BV421, clone 30-F11, Biolegend), MHCII (PerCP/Cy5.5, clone M5/114.15.2, Biolegend). Flow cytometry data were acquired using the BD FACS CANTO II (BD Biosciences) and analysed using Flowjo v10.8 software.

### Isolation of retinal CD11b + CD45 + cells for single-cell RNA sequencing 

Mice were euthanized and transcardially perfused with saline. Retinas without retinal pigment epithelium were transferred to RPMI (Gibco) containing actinomycin D (ActD, Sigma Aldrich, 30 μM) [97]. To obtain sufficient number of cells, retinas were pooled from individual mice: 32 mice for the naïve sample (32 retinas), 10 mice for ONC sample, 4 mice for ONC + P3C 4dpi sample, 4 mice for ONC + P3C 8dpi sample. The retinal samples underwent mechanical and enzymatic (collagenase I (Worthington, 10 U/ml), collagenase IV (Worthington, 400 U/ml) and DNase I (Roche, 30 U/ml) in HBSS medium (Gibco) containing ActD (Sigma Aldrich, 15 μM)) dissociation (3 × 10 min at 37 °C). Afterwards, cells were filtered, resuspended in MACS buffer (HBSS medium (Gibco) supplemented with sterile filtered EDTA (Duchefa; 2 mM) and heat-inactivated FCS (Gibco, 2%), containing ActD (Sigma Aldrich, 3 μM)) and blocked with anti-mouse CD16/CD32 (clone 2.4G2, BD Biosciences, 2 µg/µl in MACS buffer). Cells were stained with CD45-APC (30-F11, Biolegend) and CD11b-PE/Cy7 (M1/70, Biolegend) in MACS. 4′,6-diamidino-2-phenylindole (DAPI, Dako, 1 µg/ml in MACS buffer) was used to exclude dead cells and CD45^+^CD11b^+^ cells were sorted using a BD FACS ARIA III (BD Biosciences) equipped with a 100 µm nozzle. Sorted cells were collected in ME medium (RPMI medium (Gibco) supplemented with heat-inactivated FCS (Gibco, 20%), l-glutamine (Gibco, 300 μg/ml), penicillin (Gibco, 100 units/ml) and streptomycin (Gibco, 100 μg/ml), non-essential amino acids (Gibco, 1 mM), sodium pyruvate (Gibco, 1 mM), 2-mercaptoethanol (Sigma Aldrich, 0.05 mM) and ActD (Sigma Aldrich, 3 μM)) for further processing in the 10 × genomics platform.

### Single-cell RNA sequencing using 10 × genomics platform

The library construction for single-cell RNA sequencing (scRNA-seq) was performed as previously described [10]. Briefly, cellular suspensions of an estimated final concentration of 1000 cells/µl were loaded on a GemCode Single Cell Instrument (10 × Genomics) to partition them into single-cell gel beads-in-emulsion (GEM). GEMs and scRNA-seq libraries were prepared using the GemCode Single Cell 3ʹ Gel Bead and Library Kit (10 × Genomics, No. 120237) and the Chromium i7 Multiplex Kit (10 × Genomics, No. 120262) according to manufacturer’s instructions. Briefly, GEM reverse-transcription incubation was performed, followed by amplification of the full-length, barcoded cDNA, enzymatic fragmentation, library construction by 5’ adaptor attachment to generate Illumina-ready sequencing libraries and eventually sample indexing. The cDNA content of pre-fragmentation and post-sample indexing was analysed using the 2100 BioAnalyzer (Agilent). The libraries were sequenced on an Illumina HiSeq4000 flow cell with sequencing settings following the recommendations of 10 × Genomics (read 1: 26 cycles; read 2: 98 cycles; index i7: eight cycles; index i5: no cycles; 2.1 pM loading concentration).

### Alignment and quantification of gene expression in single-cell RNA sequencing data

The Cell Ranger software (10 × Genomics) v.6.0.2 was used to perform sample demultiplexing and alignment of sequencing reads to the reference genome (Mus musculus mm10), barcode processing, unique molecular identifiers filtering and single-cell 3ʹgene counting. The average of the mean reads per cell was 49,780 ± 1629 SD, with an average sequencing saturation metric of 59% ± 8% SD, as calculated by Cell Ranger. The further pre-processing and analysis of the gene expression count matrices was performed in R using Seurat v.3.2.3, DropletUtils v1.10.1.2, scater 1.18.3. The cellular barcodes, associated with low quality “empty” droplets, were filtered out using the “emptyDrops” function of the DropletUtils package with the recommended FDR cutoff ≤ 0.1 for deviation from the ambient RNA profile. The gene expression matrices were further filtered for low quality cells, normalized and scaled, followed by selection of highly variable genes, principal components analysis and clustering as previously described (Scheyltjens et al., 2022). The genes, specifically expressed in each cluster, were identified via differential expression analysis with the “FindMarkers” function of Seurat (Wilcoxon Rank Sum test). The p-values of differential expression were adjusted for multiple testing with Bonferroni correction. Clustering results were visualized using two-dimensional scatter plots with the Uniform Manifold Approximation and Projection (UMAP) method. Several of the identified clusters exhibited simultaneous expression of both macrophage and neutrophil gene markers, e.g. *C1qa, C1qb, P2ry12, Ms4a7; S100a8, S100a9, Retnlg, Csf3r*. Additionally, those clusters showed a high doublet score, as calculated by the scDblFinder package v.1.4.0, therefore they were assumed to be macrophage–neutrophil aggregates and were excluded from further analysis.

### Single-cell regulatory network inference and clustering using SCENIC

We performed single-cell regulatory network inference analysis using SCENIC v1.2.4 [46] using the raw, untransformed UMI counts as input and following the proposed workflow. The co-expression network was generated using GRNBoost2 via arboreto v0.1.5. For running GRNBoost2, the expression matrix was filtered for genes with over 30 UMI counts and expressed in at least 40 cells. The resulting transcription factor by gene targets matrix was imported in R and further analysed with the SCENIC workflow with default parameters. The regulon activity, which identifies and scores gene regulatory networks or regulons in single cells, was calculated using AUCell as previously described [46]. The better the gene targets of a regulon match the highly expressed genes of a certain cell, the higher the AUC value (also named regulon activity) of that regulon in that particular cell. The regulons were visualized in a network using Cytoscape v.3.9.1 [98].

### Modelling the intercellular communication using NicheNet

We extracted gene expression matrices of RGCs of control mice and mice 4 days post ONC using GSE137398 [50]. The gene expression data was pre-processed as described above. The clusters "41_AlphaONT", "42_AlphaOFFS", "43_AlphaONS" and "45_AlphaOFFT" were grouped as alphaRGCs, while the clusters "22_M5", "31_M2", "33_M1" and "40_M1dup" were grouped as ipRGCs. For predicting interactions between the macrophages and the RGCs, we applied the NicheNet package (v. 1.1.0), using the pre-build NicheNet prior model of ligand-receptor interactions. MDM2-7 were defined as sender, while alpha and ipRGC were defined as the receiver cell populations. Potential ligands and receptors were identified as genes, expressed in at least 10% of the sender/receiver population, respectively, and present in the prior interaction model. To prioritise the identified interactions, we performed NicheNet ligand activity analysis, which ranks the ligands based on the presence of their target genes in the gene set of interest, here defined as the differentially expressed genes in the alpha and ipRGCs between the 4dpi ONC and the naive condition (adjusted *p* value < 0.05). Next, we selected the top 40 ligands with highest ligand activity (based on the Pearson score) and added three ligands with lower ligand activity that had known neuroprotective effects (*Thbs1, Nrg1* and *Igf1*). For the selected 43 ligands, we inferred the top predicted receptors and target genes in the receiver cells. For visualising the ligand—target genes interactions, we showed the 110 most strongly predicted targets of at least one of the selected ligands, that were also part of the gene set of interest.

### Immunohistochemistry on retinal whole mounts and cryosections of retina and optic nerve

Mice were euthanized as described above and transcardially perfused with saline followed by phosphate buffered paraformaldehyde (PFA, pH 7.4, Sigma Aldrich, 4% in PBS). For retinal whole mount stainings, the eyes and subsequently the retinas were dissected, post-fixed in PFA (pH 7.4, Sigma Aldrich, 4% in PBS) for 1 h and rinsed in PBS. The retinas were incubated overnight with the primary antibody (rabbit anti-IBA1, Wako, 1/2000 diluted in PBS supplemented with pre-immune donkey serum (PID, Merck, 2%) and triton X-100 (VWR, 2%)). After rinsing in PBS, the retinas were incubated for 2 h with a donkey anti-rabbit Alexa Fluor 488 secondary antibody (DAR488, Dako, 1/200 in PBS supplemented with PID (Merck, 2%) and triton X-100 (VWR, 2%)). Mosaic pictures of the entire retinal whole mounts were made using a confocal scanning microscope (Olympus FV 1000D). Microglia density, soma size and roundness were analysed using a spatial statistics approach, all as previously described [99].

For retinal or optic nerve cryosections, complete eyes and optic nerves were dissected, postfixed for 1 h at room temperature and cryoprotected through an ascending series of sucrose (Sigma Aldrich, 10%–20%–30% in PBS). Afterwards, eyes or optic nerves were embedded in TissueTek (Sakura) and 14 µm thick sagittal sections of the eyes or longitudinal optic nerve sections were made. For immunolabeling of the cryosections of the eyes, epitope retrieval was accomplished using citrate buffer (pH 6, citric acid (Chem-lab, 10 mM) and Tween 20 (Sigma Aldrich, 0.05%) in H_2_O). Aspecific binding places were saturated with PID (Merck, 20%) in Tris-sodium chloride blocking buffer (TNB, triton X-100 (VWR, 1.5 mM %), Tris-HCl (Acros Organics, 0.1 M), NaCl (Fischer Scientific, 150 mM) and blocking reagent (Perkin Elmer, 0.5%) in PBS)) and the primary antibodies (chicken anti-GFP, Abcam, 1/500 in TNB and rabbit anti-IBA1, Wako, 1/2000 in TNB) were incubated overnight at room temperature. After rinsing, the slides were incubated with, respectively, donkey anti-chicken Alexa Fluor 488 (DACh488, Dako,1/200 in TNB) and donkey anti-rabbit biotin secondary antibody (DARbiotin, Dako, 1/300 in TNB), followed by subsequent incubation with streptavidin-horse radish peroxidase (Strep-HRP, Dako, 1/100 in TNB) and tyramid signal amplification (TSA Cy3-Tyr, Thermofisher Scientific, 1/50 in amplification buffer). Finally, the slides were counterstained with DAPI (Dako, 1 µg/ml in PBS). Images of the mid-sagittal retinal cryosections were taken using a Leica DM6 (Olympus) fluorescent microscope. For the optic nerves, images of mid-longitudinal sections that contained the ONC site were taken using a confocal scanning microscope (Olympus FV 1000D).

### Quantification of axonal growth

Axon growth was quantified on three mid-longitudinal cryosections of the optic nerve by manually counting the number of CTB^+^ axons every 150 µm (distance d) beyond the crush site, using ImageJ [100]. In addition, at each distance, the cross-sectional width of the nerve was measured along. The total estimated number of axons in the optic nerve extending distance d from the ONC lesion site was calculated using following formula where the radius of the optic nerve was set at *r* = 150 µm and the thickness of the sections was t = 14 µm, all as described previously [101].

$$\Sigma a_{d} = \pi r^{2} . \frac{{{\text{Average}} \left( {\# {\text{axons}}/{\mu m}\;{\text{of}}\;{\text{nerve}}\;{\text{width}}} \right)}}{t}$$

The results obtained for each of the three sections per nerve were averaged.

### Statistics

Statistical analyses were performed using GraphPad Prism 8 software (GraphPad Software). Normal distribution was evaluated using a Kolmogorov–Smirnov test and parallel equal variance between groups was tested. Outliers were identified and excluded based on a Grubb’s test (extreme studentised deviate method). The values are expressed as mean values ± standard error (SEM). Statistical tests are specified in the figure legends, together with the number of biologically independent samples (*n*). Statistically significant differences between multiple groups are specified using different letters. Conditions with the same letter are not significantly different, while conditions with different letters are significantly different from each other. Statistical significance between two groups were specified with **** for *p* < 0.0001, *** for *p* < 0.001, ** for *p* < 0.01, and * for *p* < 0.5.

## Supplementary Information

**Additional file 1.** scRNA-seq of CD45^+^CD11b^+^ cells from naïve or ONC retinas. **A** UMAP showing all CD11b^+^ cells profiled from the healthy and ONC retinas. BAM, border associated macrophage, cDC: conventional dendritic cell, migDC: migratory dendritic cell, MDM: monocyte-derived macrophage, Mg: microglia, MO, monocyte, N: neutrophils, NK: natural killer cell. **B** UMAPs showing the expression of the indicated genes. Red line highlights the putative macrophage-neutrophil doublets. **C** Volcano plot displaying differential expression between Mg3 and Mg1. Genes with adjusted p-value <0.01 and I Log2I >1 are shown in red. **D** Volcano plot displaying differential expression between Mg4 and Mg1. Genes with adjusted p-value <0.01 and I Log2I >1 are shown in red. **E** Quantification of the density and activityof microglia in retina at different timepoints after ONC corresponding with images shown in figure 1E. Data are shown as mean ± SEM. Repeated measures one-way ANOVA followed by Tukey’s multiple comparisons test, statistical significance between different timepoints is indicated using different letters: conditions that share the same letter are not significantly different, while conditions with different letters are significantly different from each other. n=3-4 mice per condition. **F** UMAPs showing the expression of the indicated genes, corresponding to the dataset shown in S1A.

**Additional file 2.** Inflammatory treatment stimulates axonal initiation. **A** Representative images of longitudinal cryosections of the optic nerve showing regenerating axons that were CTB-traced at different timepoints after ONC and ONC+P3C. The ONC site is indicated by an asterisk. Scale bar 50µm. **B** Quantification of axonal regeneration in the optic nerve of mice at different timepoints after ONC or ONC combined with P3C treatment. The number of regrowing axons was analysed at various distances starting at 150 µm from the ONC lesion site. Representative images of n = 3 mice per condition. Quantitative data after ONC+IS are shown as mean ± SEM. Repeated measures two-way ANOVA followed by Tukey’s multiple comparisons test, statistical significance between different conditions at the same distance is indicated with different letters, n=3-5 mice per condition.

**Additional file 3.** Full scRNA-seq dataset of naïve, ONC and ONC+P3C CD11b^+^CD45^+^ cells. **A** UMAP and cluster annotation showing 22081 cells of both healthy, injuredand regeneratingretinas. BAM, border associated macrophage, cDC: conventional dendritic cell, migDC: migratory dendritic cell, MDM: monocyte-derived macrophage, Mg: microglia, MO, monocyte, N: neutrophils, NK: natural killer cell. **B**,**C** UMAP showing 6997 cells of retinas at 4dpi ONC+P3Cand 7956 cells of retinas at 8dpi ONC+P3C. Individual pie charts show the distribution of neutrophils, monocytes or alle immune populations. Numbers in the pie chart are percentages of the cells from the corresponding cluster.

**Additional file 4.** Expression of pro-regenerative genes in cluster MDM7. Gene ontology analysis on the upregulated genes in Mg5 versus Mg2> 20; log2>1) showing the top 20 enriched GO terms for Mg5.

**Additional file 5.** Nichenet analysis of MDMs against injured RGCs. **A** Overview of potential receptors on the retinal ganglion cells of the ligands expressed by the different macrophage clusters. The colourrepresents the regulatory potential of the receptors based on the prior model of ligand-receptor interactions.Receptor expression in the different retinal ganglion cell populations is shown with the colourrepresenting the scaled average expression in the corresponding cluster. **B** Overview of the predicted target genes in the retinal ganglion cells of the ligands expressed by the different macrophage clusters. The colourrepresents the regulatory potential of the target genes based on the prior model of ligand-target gene interactions. **C** Circle plot of potential ligand-receptor pairs. It shows the links between predicted ligands from the different monocyte-derived macrophage clusters of the regenerating retinawith their associated receptors found on alpha- and intrinsically photosensitive retinal ganglion cells.

**Additional file 6.** Pro-regenerative gene signature in cluster MDM7. **A** Corresponding dot plot of the recruited monocyte-derived macrophage populations showing the expression of selected pro-regenerative genes, with the dot size representing the percentage of cells expressing the gene and the colour representing its average expression within a cluster. **B** UMAP plots showing expression of the indicated genes, *Cxcl12* and *Ocm*, corresponding to the dataset shown in S3A.

**Additional file 7**. Overview of mouse strains used in this study.

## Data Availability

The scRNA-seq datasets described in this article will be made accessible through our interactive webserver at www.brainimmuneatlas.org. Furthermore, all raw data and gene-cell count matrices are deposited at GEO (NCBI) with accession code GSE232470.
