# Supplementary figures and images for: Immune stimulation recruits a subset of pro-regenerative macrophages to the retina that promotes axonal regrowth of injured neurons
Source: Acta Neuropathol Commun. 2023 May 24;11:85. doi: 10.1186/s40478-023-01580-3 (PMC10210300; doi:10.1186/s40478-023-01580-3)

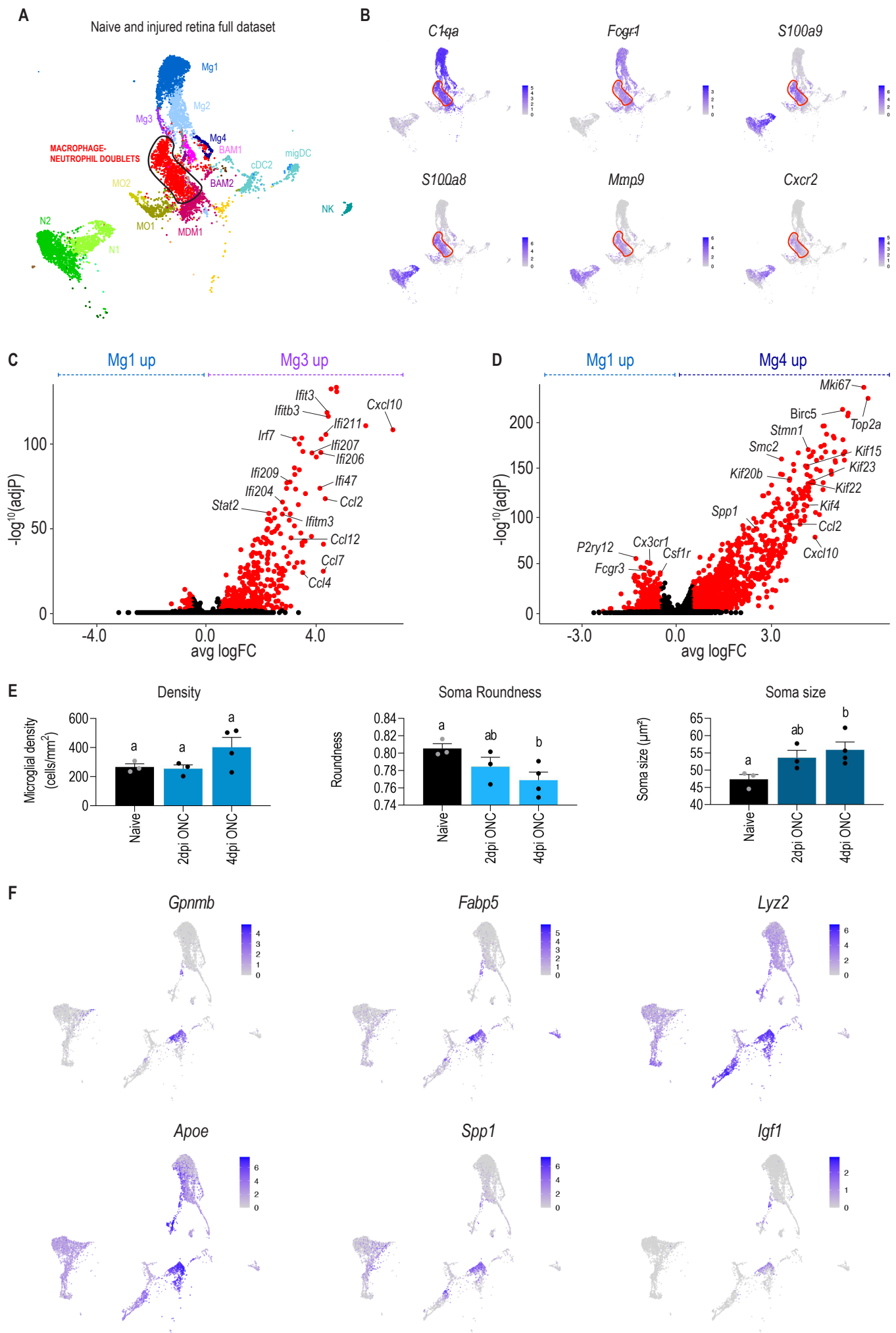

FIGURE S1

Supplement: Supplementary file 1 — Additional file 1. scRNA-seq of CD45+CD11b+ cells from naïve or ONC retinas. A UMAP showing all CD11b+ cells profiled from the healthy and ONC retinas. BAM, border associated macrophage, cDC: conventional dendritic cell, migDC: migratory dendritic cell, MDM: monocyte-derived macrophage, Mg: microglia, MO, monocyte, N: neutrophils, NK: natural killer cell. B UMAPs showing the expression of the indicated genes. Red line highlights the putative macrophage-neutrophil doublets. C Volcano plot displaying differential expression between Mg3 and Mg1. Genes with adjusted p-value <0.01 and I Log2I >1 are shown in red. D Volcano plot displaying differential expression between Mg4 and Mg1. Genes with adjusted p-value <0.01 and I Log2I >1 are shown in red. E Quantification of the density and activityof microglia in retina at different timepoints after ONC corresponding with images shown in figure 1E. Data are shown as mean ± SEM. Repeated measures one-way ANOVA followed by Tukey’s multiple comparisons test, statistical significance between different timepoints is indicated using different letters: conditions that share the same letter are not significantly different, while conditions with different letters are significantly different from each other. n=3-4 mice per condition. F UMAPs showing the expression of the indicated genes, corresponding to the dataset shown in S1A. [file 40478_2023_1580_MOESM1_ESM.pdf]

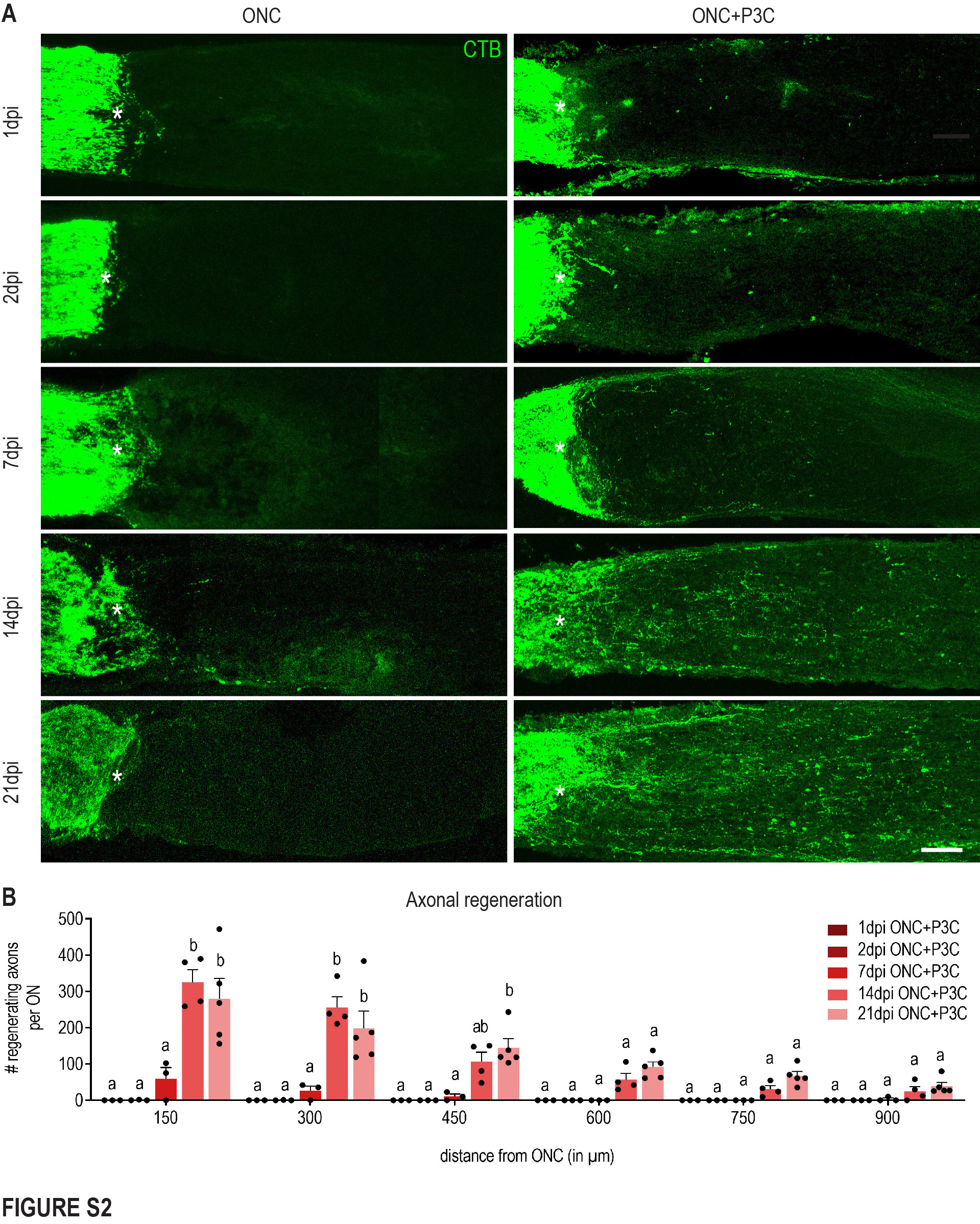

Supplement: Supplementary file 2 — Additional file 2. Inflammatory treatment stimulates axonal initiation. A Representative images of longitudinal cryosections of the optic nerve showing regenerating axons that were CTB-traced at different timepoints after ONC and ONC+P3C. The ONC site is indicated by an asterisk. Scale bar 50µm. B Quantification of axonal regeneration in the optic nerve of mice at different timepoints after ONC or ONC combined with P3C treatment. The number of regrowing axons was analysed at various distances starting at 150 µm from the ONC lesion site. Representative images of n = 3 mice per condition. Quantitative data after ONC+IS are shown as mean ± SEM. Repeated measures two-way ANOVA followed by Tukey’s multiple comparisons test, statistical significance between different conditions at the same distance is indicated with different letters, n=3-5 mice per condition. [file 40478_2023_1580_MOESM2_ESM.jpg]

**A**

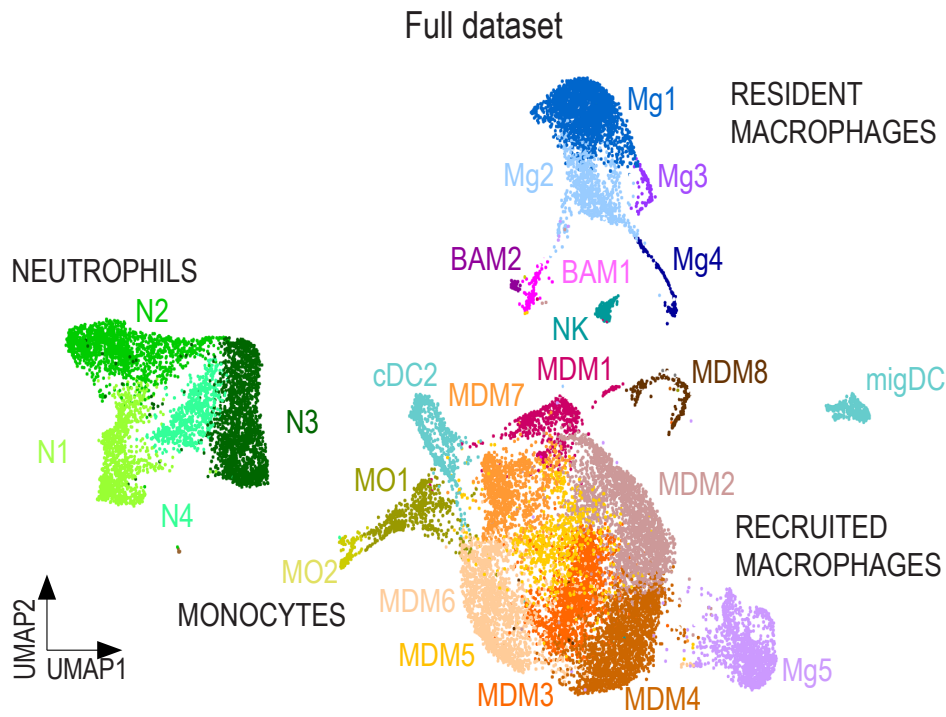

**B**

Regenerating (4dpi ONC+P3C)

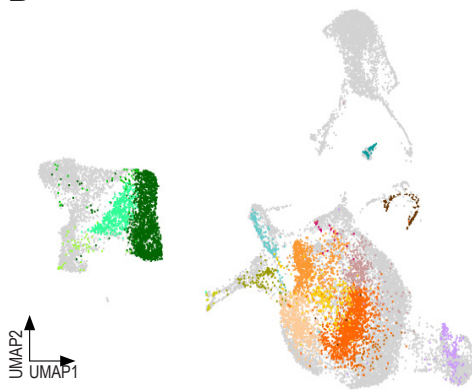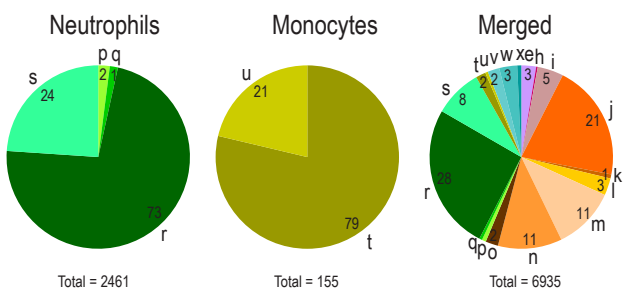

**C**

Regenerating (8dpi ONC+P3C)

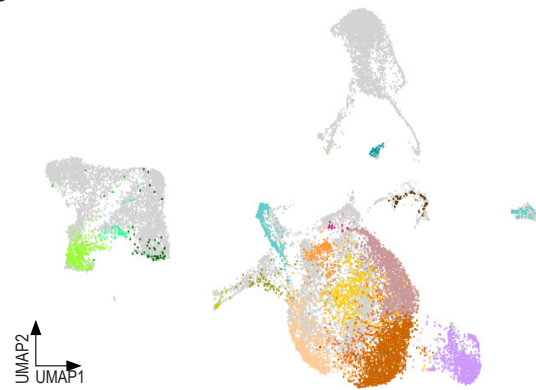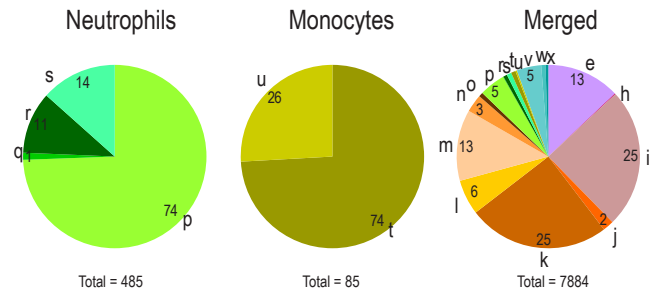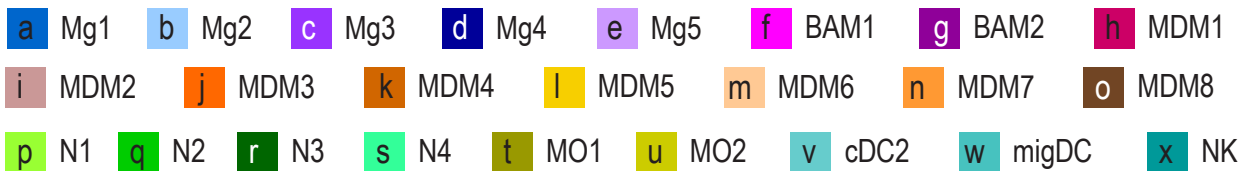

**FIGURE S3**

Supplement: Supplementary file 3 — Additional file 3. Full scRNA-seq dataset of naïve, ONC and ONC+P3C CD11b+CD45+ cells. A UMAP and cluster annotation showing 22081 cells of both healthy, injuredand regeneratingretinas. BAM, border associated macrophage, cDC: conventional dendritic cell, migDC: migratory dendritic cell, MDM: monocyte-derived macrophage, Mg: microglia, MO, monocyte, N: neutrophils, NK: natural killer cell. B,C UMAP showing 6997 cells of retinas at 4dpi ONC+P3Cand 7956 cells of retinas at 8dpi ONC+P3C. Individual pie charts show the distribution of neutrophils, monocytes or alle immune populations. Numbers in the pie chart are percentages of the cells from the corresponding cluster. [file 40478_2023_1580_MOESM3_ESM.pdf]

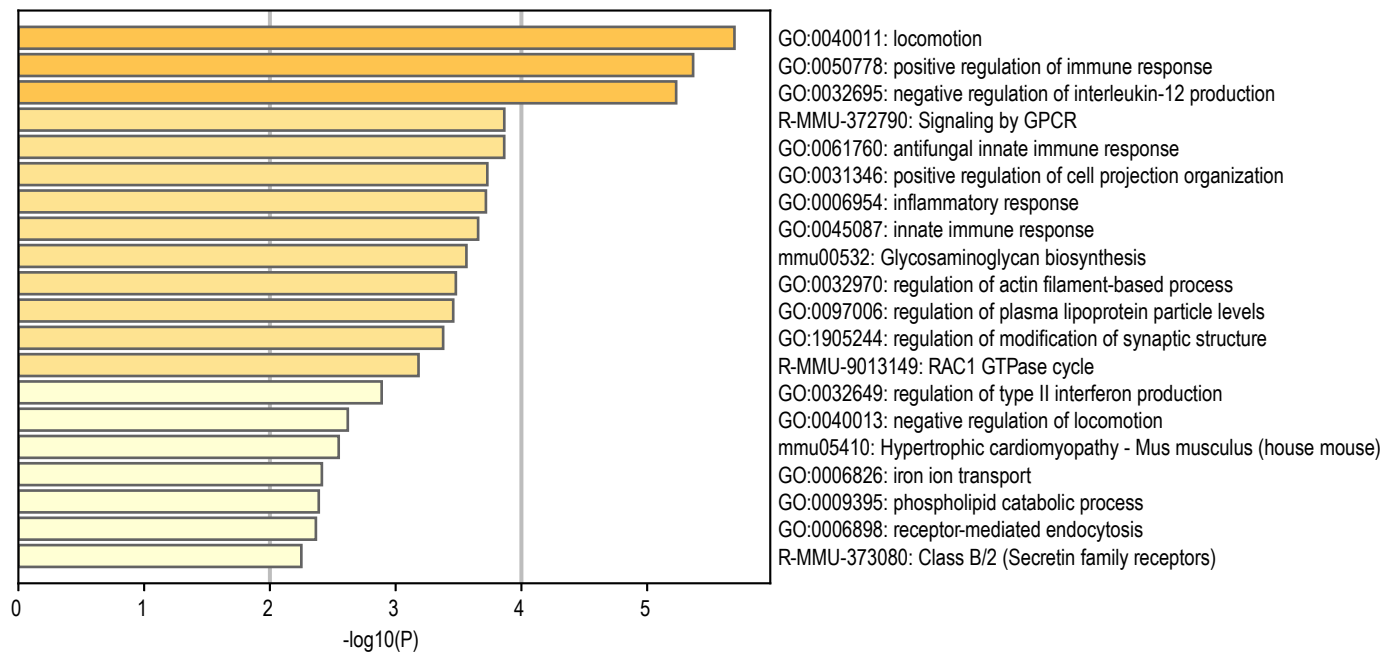

**FIGURE S4**

Supplement: Supplementary file 4 — Additional file 4. Expression of pro-regenerative genes in cluster MDM7. Gene ontology analysis on the upregulated genes in Mg5 versus Mg2> 20; log2>1) showing the top 20 enriched GO terms for Mg5. [file 40478_2023_1580_MOESM4_ESM.pdf]

A

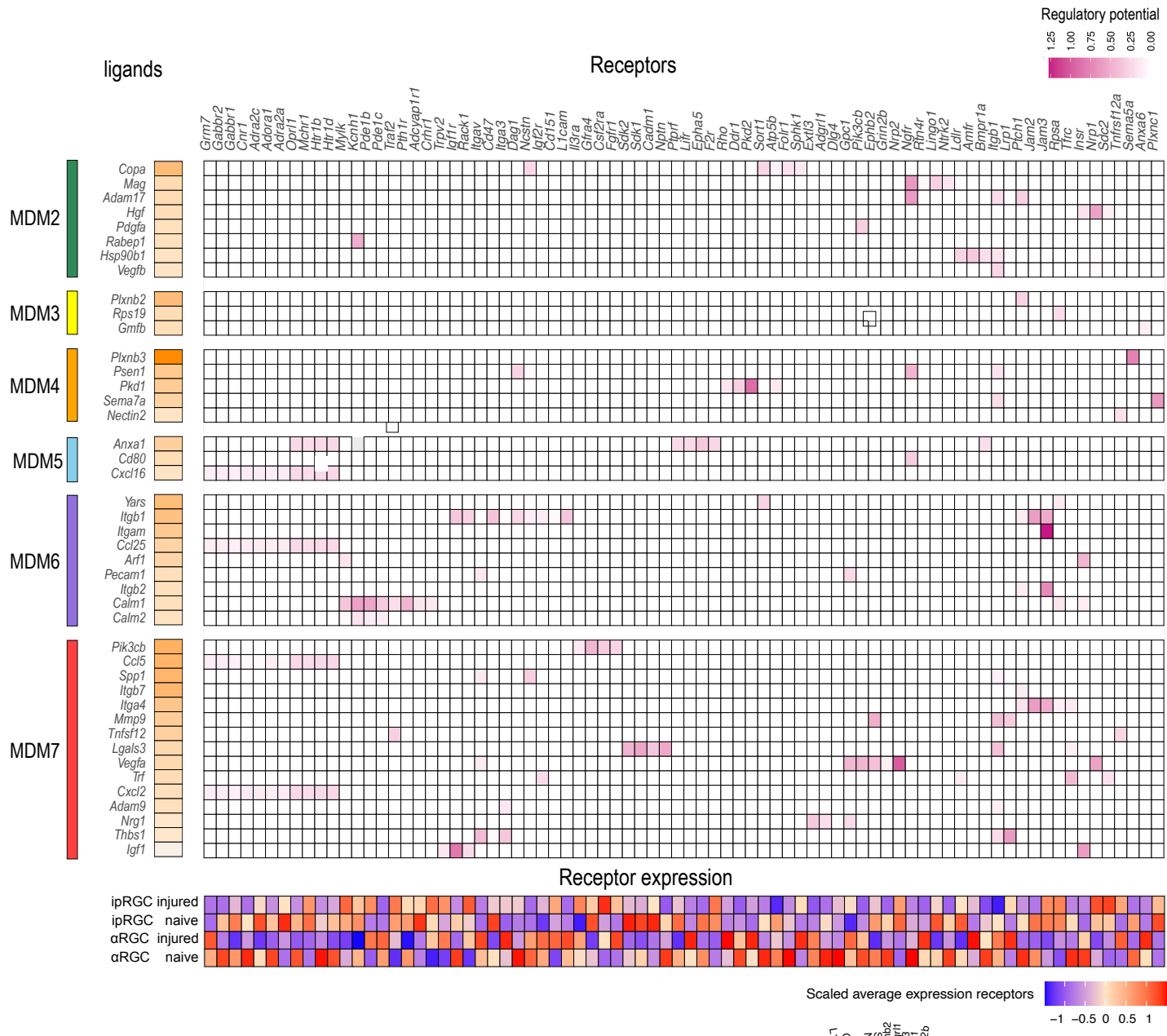

B

Predicted target genes

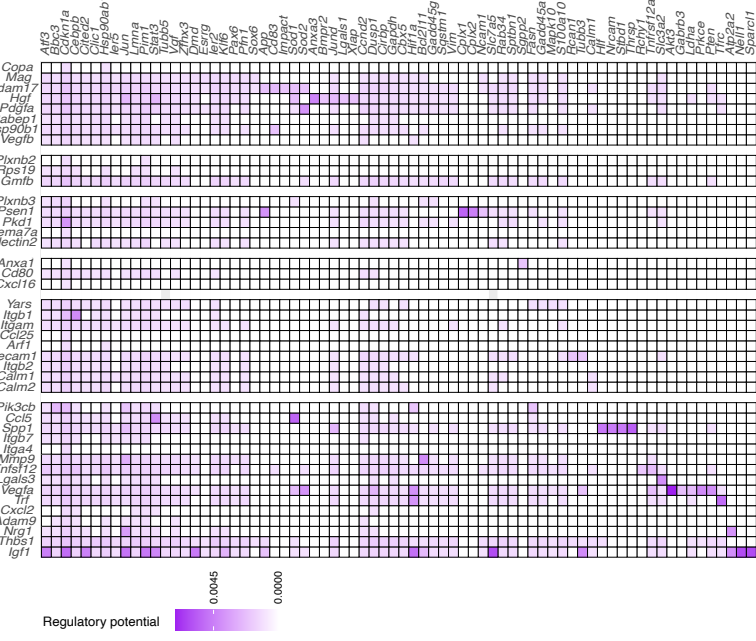

C

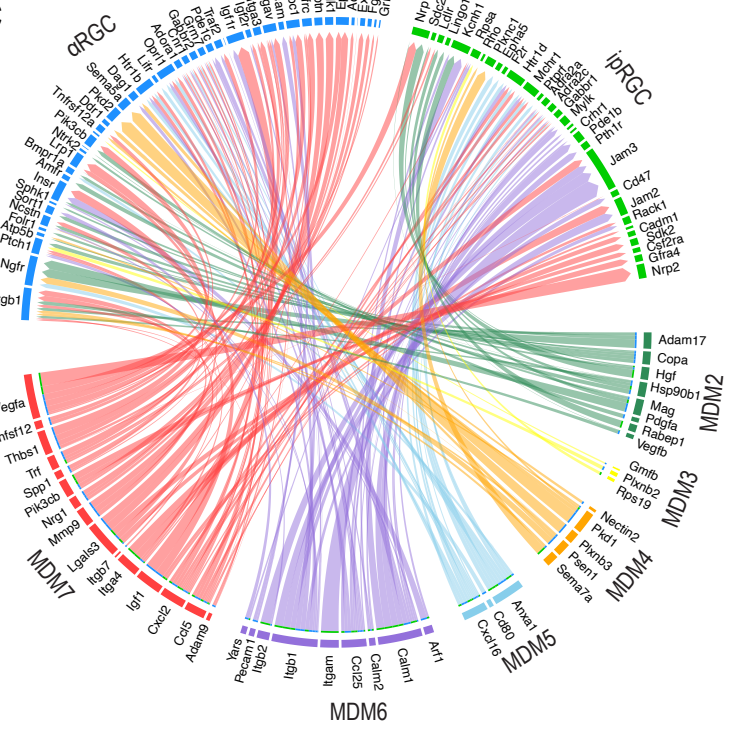

FIGURE S5

Supplement: Supplementary file 5 — Additional file 5. Nichenet analysis of MDMs against injured RGCs. A Overview of potential receptors on the retinal ganglion cells of the ligands expressed by the different macrophage clusters. The colourrepresents the regulatory potential of the receptors based on the prior model of ligand-receptor interactions.Receptor expression in the different retinal ganglion cell populations is shown with the colourrepresenting the scaled average expression in the corresponding cluster. B Overview of the predicted target genes in the retinal ganglion cells of the ligands expressed by the different macrophage clusters. The colourrepresents the regulatory potential of the target genes based on the prior model of ligand-target gene interactions. C Circle plot of potential ligand-receptor pairs. It shows the links between predicted ligands from the different monocyte-derived macrophage clusters of the regenerating retinawith their associated receptors found on alpha- and intrinsically photosensitive retinal ganglion cells. [file 40478_2023_1580_MOESM5_ESM.pdf]

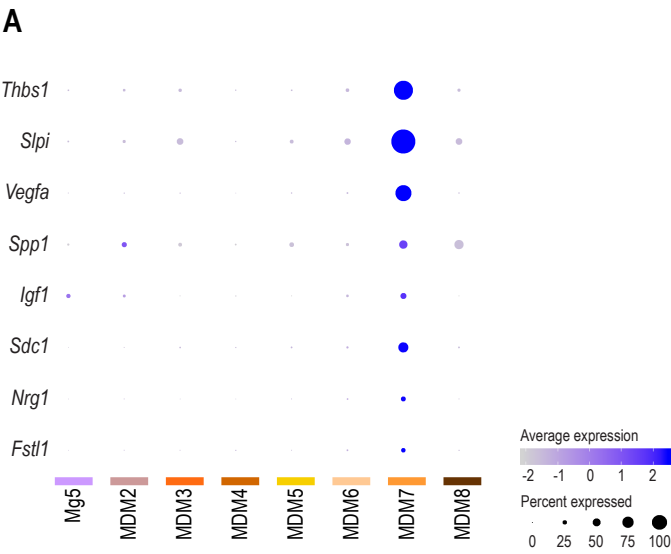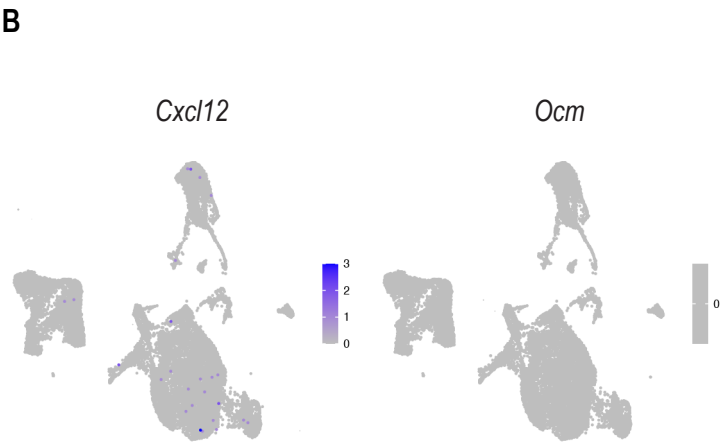

FIGURE S6

Supplement: Supplementary file 6 — Additional file 6. Pro-regenerative gene signature in cluster MDM7. A Corresponding dot plot of the recruited monocyte-derived macrophage populations showing the expression of selected pro-regenerative genes, with the dot size representing the percentage of cells expressing the gene and the colour representing its average expression within a cluster. B UMAP plots showing expression of the indicated genes, Cxcl12 and Ocm, corresponding to the dataset shown in S3A. [file 40478_2023_1580_MOESM6_ESM.pdf]
