# Supplementary material for: Immune stimulation recruits a subset of pro-regenerative macrophages to the retina that promotes axonal regrowth of injured neurons
Source: Acta Neuropathol Commun. 2023 May 24;11:85. doi: 10.1186/s40478-023-01580-3 (PMC10210300; doi:10.1186/s40478-023-01580-3)
Supplement: Supplementary file 7 — Additional file 7. Overview of mouse strains used in this study. [file 40478_2023_1580_MOESM7_ESM.docx]

| Strain | Reference | Genetic background | Genotype of used mice | Total number | Sex |
| --- | --- | --- | --- | --- | --- |
| C57BL/6J |  | C57BL/6J | N/A | 241 | M & F (± equal ratio) |
| Lyz2-GFP | 90 | C57BL/6J | *Lyz2*^GFP/GFP^ | 27 | M & F (± equal ratio) |
| CCR2-/- | 45 | C57BL/6J | *CCR2*^-/-^ | 17 | M & F (± equal ratio) |
| Cx3cr1^CreER^ : R26-YFP | 91 and 92 | C57BL/6J | *Cx3cr1*^CreER/+^ : *R26*^YFP/+^ | 40 | M & F (± equal ratio) |

**Table S1.** Overview of mouse strains used in this study.
